# A comparison of classical and machine learning-based phenotype prediction methods on simulated data and three plant species

**DOI:** 10.3389/fpls.2022.932512

**Published:** 2022-11-04

**Authors:** Maura John, Florian Haselbeck, Rupashree Dass, Christoph Malisi, Patrizia Ricca, Christian Dreischer, Sebastian J. Schultheiss, Dominik G. Grimm

**Affiliations:** ^1^ Technical University of Munich, Campus Straubing for Biotechnology and Sustainability, Bioinformatics, Straubing, Germany; ^2^ Weihenstephan-Triesdorf University of Applied Sciences, Bioinformatics, Straubing, Germany; ^3^ Computomics GmbH, Tübingen, Germany; ^4^ Technical University of Munich, Department of Informatics, Garching, Germany

**Keywords:** phenotype prediction, genomic selection, plant phenotyping, machine learning, *Arabidopsis thaliana*

## Abstract

Genomic selection is an integral tool for breeders to accurately select plants directly from genotype data leading to faster and more resource-efficient breeding programs. Several prediction methods have been established in the last few years. These range from classical linear mixed models to complex non-linear machine learning approaches, such as Support Vector Regression, and modern deep learning-based architectures. Many of these methods have been extensively evaluated on different crop species with varying outcomes. In this work, our aim is to systematically compare 12 different phenotype prediction models, including basic genomic selection methods to more advanced deep learning-based techniques. More importantly, we assess the performance of these models on simulated phenotype data as well as on real-world data from *Arabidopsis thaliana* and two breeding datasets from soy and corn. The synthetic phenotypic data allow us to analyze all prediction models and especially the selected markers under controlled and predefined settings. We show that Bayes B and linear regression models with sparsity constraints perform best under different simulation settings with respect to explained variance. Further, we can confirm results from other studies that there is no superiority of more complex neural network-based architectures for phenotype prediction compared to well-established methods. However, on real-world data, for which several prediction models yield comparable results with slight advantages for Elastic Net, this picture is less clear, suggesting that there is a lot of room for future research.

## 1 Introduction

Currently, the agricultural industry is under great pressure to deliver new crop varieties quickly for a changing climate and a fewer resource use. Genomic selection (GS) offers breeders the ability to accurately select plants with the potentially highest return on investment for the traits they are interested in, such as yield and drought tolerance, directly from genotype data (Asseng et al., 2015; Parent et al., 2018). By using GS, crosses can be planned with precision, leading to faster and more resource-efficient breeding programs. For these reasons, modern plant breeding programs rely on GS to guide the development of improved crops (R2D2 Consortium et al., 2021). GS uses genome-wide markers to predict phenotypes or breeding values and was first proposed by Meuwissen et al. (2001). Since then, several prediction methods have been established and used in animal as well as plant breeding (Hayes et al., 2009; Gianola and Rosa, 2015; Heslot et al., 2015; Arruda et al., 2016). Commonly used statistical methods are linear mixed models, such as the so-called Best Linear Unbiased Predictor (BLUP) and its variations, e.g., genomic BLUP (GBLUP) or Ridge-Regression BLUP (RR-BLUP) (Meuwissen et al., 2001), and Bayesian linear regression models, also known as the Bayesian alphabet (Meuwissen et al., 2001; Gianola et al., 2009). GBLUP uses a genomic relationship matrix calculated from genetic markers. All marker information is incorporated in these models, and their model coefficients are assumed to be normally distributed and to explain an equal amount of variance. RR-BLUP also uses genomic information but additionally implements a penalizing function to equally shrink the markers’ coefficients. The shrinkage factor is estimated to be the ratio of the residual variance and the variance of the regression coefficients of the marker effects. Under certain conditions, RR-BLUP has been proven to be mathematically equivalent to GBLUP (Goddard, 2008; Hayes et al., 2009).

Even though these models only consider additive effects, they show a good performance for a variety of species and traits and are considered as state-of-the-art in GS (Ma et al., 2018; Molenaar et al., 2018). In order to capture more complex genetic architectures, several machine learning (ML) approaches were included in phenotype prediction studies (Azodi et al., 2019). For instance, several researchers employed regularized linear regression models, e.g., in combination with L1-regularization for an implicit feature selection (Mcdowell, 2016; Liu et al., 2019). Furthermore, kernel-based techniques, e.g., Support Vector Machines (SVMs) respective Support Vector Regression (SVR), were often included in phenotype prediction studies. Beyond that, ensemble-based methods, such as Random Forests (RF) and gradient boosting, e.g., XGBoost (XGB), are widely applied in research and industry (Azodi et al., 2019; Crossa et al., 2019; Liu et al., 2019; Montesinos-López et al., 2019). Recently, several studies regarding deep learning (DL) approaches for phenotype prediction in animals (Gianola et al., 2011; Abdollahi-Arpanahi et al., 2020), plants (Ma et al., 2018; Montesinos-López et al., 2018a; Azodi et al., 2019), and even humans (Bellot et al., 2018) have been published with varying results. In an early work, Mcdowell (2016) applied feedforward Multilayer Perceptrons (MLPs) for the prediction of different phenotypes in *Arabidopsis thaliana*. In their study, the neural networks performed similarly compared to linear regression-based approaches. Ma et al. (2018) showed that Convolutional Neural Networks (CNNs) performed better or at least comparable to BLUP-based methods in several wheat traits. For complex traits in Holstein bulls, DL-based approaches were outperformed by XGB (Abdollahi-Arpanahi et al., 2020). Azodi et al. (2019) compared CNNs and MLPs with RR-BLUP as well as additional statistical and classical ML approaches for several traits in six different plant species. In their comparison, there is no predominant model for all phenotypes and species. However, they concluded that linear models perform well on all kinds of traits, whereas the performance of non-linear algorithms varies depending on the trait and number of available samples. Montesinos-López et al. (2018a) compared MLP architectures and GBLUP for maize and wheat datasets, where they considered models with and without genotype–environment interaction. They showed that GBLUP performs best for most considered traits when taking the interaction between the genotype and environment into account. In contrast, DL-based approaches outperformed GBLUP in six out of nine traits when ignoring the environment interaction. Furthermore, Sandhu et al. (2020) showed that both CNNs and MLPs outperformed RR-BLUP in five different traits in spring wheat, when considering different years of planting. Additionally, with respect to multi-trait GS models, Sandhu et al. (2021) showed that Random Forests and MLPs outperform GBLUP and Bayesian alphabet methods. Pook et al. (2020) tried to capture the influence of marker variants in different genomic regions by using Local Convolutional Neural Networks (LCNNs) with a region-specific filter. Although LCNNs outperformed CNNs and MLPs on several *Arabidopsis thaliana* traits, GBLUP still delivered better results. However, with an increasing number of samples, the advantage of GBLUP decreased. In summary, as long as the availability of genomic and phenotypic data in plants is still limited, there is no consistent evidence on advantages using DL-based techniques (Montesinos-López et al., 2018a; Azodi et al., 2019; Pook et al., 2020). Although several studies showed the advantage of artificial neural nets for prediction tasks (Montesinos-López et al., 2018a; Sandhu et al., 2020), the results are hardly comparable. The studies were performed on different species with different traits (Montesinos-López et al., 2018a; Azodi et al., 2019; Pook et al., 2020; Sandhu et al., 2020), varying ways of evaluation and hyperparameter optimization, and different feature sets, e.g., due to the inclusion of environmental effects (Montesinos-López et al., 2018a; Sandhu et al., 2020) or multi-trait predictions (Montesinos-López et al., 2018b; Sandhu et al., 2021).

In this work, we show a systematic comparison of 12 phenotype prediction models. For that purpose, we use both a variety of synthetic as well as real-world data, including the model organism *Arabidopsis thaliana* and two real breeding datasets from soy and corn. Regarding phenotype prediction models, we include RR-BLUP and models from the Bayesian alphabet, classical ML-based methods, such as regularized linear regression and SVR as well as two ML-based ensemble learners and three different neural network architectures. Consequently, we are able to evaluate the behavior of different types of phenotype prediction models under distinct circumstances. Beyond that, we employ Bayesian Optimization for hyperparameter selection of the prediction models. This is a state-of-the-art approach in ML but with limited spread in plant phenotype prediction (Bergstra et al., 2011; Turner et al., 2021; Westhues et al., 2021). By doing so, we minimize a potential bias of our study due to not ideal hyperparameters, which might especially impede the performance of ML-based approaches with a higher number of parameters. A focus of this research is on the analysis of phenotype prediction based on synthetic data. In contrast to real-world data, synthetic data enable the analysis under predefined settings. Beyond that, we analyze the single-nucleotide polymorphisms (SNPs) that were considered important by prediction models in comparison with effect sizes of synthetic data and results of genome-wide association studies (GWAS) for real-world data.

The remainder of this paper is organized as follows: In Section 2, we describe the material and methods. Then, we outline and discuss our results in Section 3. In Section 4, we draw conclusions and give a future outlook.

## 2 Materials and methods

In the following section, we first summarize the real-world data used for our analyses and describe the method used for generating synthetic phenotypes. Next, we outline the different phenotype prediction models that we used for our comparative study as well as the experimental settings. Finally, we give details on how we analyzed the importance of selected features.

### 2.1 Real-world data

We evaluated the performance of different classical and machine learning-based models for phenotype prediction on simulated data as well as on three different plant species, including inbred data from the model organism *Arabidopsis thaliana* and two real-world datasets from plant breeders. Dataset statistics for all phenotypes, such as the number of samples and SNPs, as well as histograms showing the distribution of the phenotype values can be found in the Supplementary (**Supplementary Table S1** and **Figure S1**).

#### 2.1.1 *Arabidopsis thaliana*

As genotypic data, a fully imputed SNP matrix with 2,029 samples and about three million homozygous SNPs was used (Arouisse et al., 2020). On that data, we applied a linkage-disequilibrium (LD) pruning using PLINK v1.9 to exclude highly correlated markers (Purcell et al., 2007; Chang et al., 2015). Phenotypic data was downloaded from the publicly available AraPheno database (Seren et al., 2016; Togninalli et al., 2020). We used four different quantitative traits from the 1001 Genomes Project in *Arabidopsis thaliana* (The 1001 Genomes Consortium, 2016): the flowering time at 10°C (FT10, https://www.doi.org/10.21958/phenotype:261), the days to flowering (DTF1, https://www.doi.org/10.21958/phenotype:703), the rosette leaf number (RL, https://www.doi.org/10.21958/phenotype:704), and the flower diameter (Diameter, https://www.doi.org/10.21958/phenotype:707). After matching of the genotypic and phenotypic data, we filtered the SNP matrix for duplicates, i.e., markers which are identical after encoding, and excluded all SNPs with a minor allele frequency of less than 10%. Thus, between 44k and 67k markers and between 656 and 1,058 samples remained per phenotype (see **Supplementary Table S1** for the exact numbers).

#### 2.1.2 Corn

The first dataset of our commercial plant breeding partners contains genotype and phenotype data for 1,411, respective 1,797 unique samples of hybrid corn from the same environment (i.e., the same test field location and planting season). Phenotype values for yield and percentage water content (PWC) were observed for each hybrid, while the genotypes were determined for the parental lines. The genotype data consists of 8,708 chip-based SNP markers from 870 female and 569 male parental lines. The parents are a mix of doubled haploid homozygous lines and inbred F4/F5 lines that contain some heterozygous loci. Genotypes of the hybrids were constructed *in silico* from the corresponding parental combination using the ×SeedScore^®^ (Computomics) technology. After matching the samples and filtering for duplicates, approximately 8k markers remained.

#### 2.1.3 Soy

The second breeding dataset contains approximately 500 unique parental lines from a soy line breeding program consisting of 810 genetic markers each. The parental lines are after several generations of selfing and are therefore quite homogeneous. All lines were growing in the same environment as well. The markers were selected based on a quantitative trait locus (QTL) analysis and show a high correlation with the traits of interest. Phenotype values for yield, maturation group (MatG), and plant height (Height) were observed for each parent. After matching the samples and filtering for duplicate SNPs, approximately 600 markers remained.

### 2.2 Synthetic data

Additionally, we simulated artificial phenotypes with different configurations using real homozygous *Arabidopsis thaliana* markers from the LD-pruned genotype matrix described above. We removed all duplicated markers (markers that are identical after encoding) and markers with no variation. Then, we randomly sampled 10,000 SNPs across all chromosomes with a minor allele frequency greater than 10%. The resulting genotype matrix was used both for the simulation of artificial phenotypes and as input of the different prediction models. For the simulation of artificial phenotypes, we employed the following underlying linear mixed model (LMM). Let *n* be the number of samples, *k* the number of fixed effects, and *b* the number of random effects. Then,


(1) y=Xβ+u+ϵ,

where y *∈* ℝ*^n^* denotes the vector of phenotyic values, ***X** ∈* ℝ*^n*×*k^* is the design matrix of the fixed effects ***β** ∈* ℝ^k^, and u :=***Zγ*** denotes the random effects (i.e., the genetic similarity between the samples), where ***Z** ∈ ℝ ^n*×*b^* is the design matrix of the random effects and the parameters ***γ**  ∈ ℝ ^b^* follow a Gaussian distribution. Further, ***ϵ** ∈ ℝ ^n^* is a vector of residuals following a known probability distribution.

First, we randomly chose *n* samples from the genotype matrix and randomly selected 1,000 markers across all chromosomes to simulate the polygenic background (i.e., the design matrix of the random effects). The corresponding parameters ***γ*** were drawn from a Gaussian distribution with zero mean and a standard deviation of 0.1. Then, we drew random noise, either from a normal distribution or, for skewed phenotypes, from a gamma distribution. This was parameterized so that the random effect explained a fixed amount *h* of the phenotypic variance *via* the following formula:


(2) $$\sigma^{2}=Var\left(\epsilon \right)=\frac{1-h}{h}Var\left(u\right).$$

To adjust the skewness, we additionally considered different shape parameters for the gamma distribution. Finally, we added one or several fixed effects ***x**_i_* in a pure additive manner or with additional multiplicative effects, to explain a ratio of about *c*=0.3 of the total phenotypic variance. For this, we computed the parameters *via*


(3) $$\beta_{i}=\sqrt{\frac{c}{1-c}\cdot \frac{Var\left(\text{y}\right)}{Var\left({\text{x}}_{i}\right)}}.$$

To analyze different scenarios, we simulated various phenotypes for three different heritabilities (*h=*0.7, *h*=0.85, *h*=0.95). For this purpose, we created 12 different configurations, as summarized in **Table 1**. For better readability, the simulations are subsequently named as follows: *A* (#100), *B* (#500), *C* (#1000), *D* (#2000), *E* (MultWeak), *F* (MultStrong), *G* (SkewedWeak), *H* (SkewedStrong), *I* (Add5), *J* (Add20), *K* (Add50), *L* (Add100).

**Table 1:** Overview of simulation settings to create synthetic phenotypes for real *Arabidopsis thaliana* genotypes.

Sim	*n*	*k*	*y*	*c*	*ϵ*
*A*	100	1	*β*_1_x_1_ *+ u + **ϵ***	*c*_1_ = 0.3	*𝒩 * (0, σ^2^)
*B*	500	1	*β*_1_x_1_ *+ u + **ϵ***	*c*_1_ = 0.3	*𝒩 * (0, σ^2^)
*C*	1,000	1	*β*_1_x_1_ *+ u + **ϵ***	*c*_1_ = 0.3	*𝒩 * (0, σ^2^)
*D*	2,000	1	*β*_1_x_1_ *+ u + ***ϵ****	*c*_1_ = 0.3	*𝒩 * (0, σ^2^)
*E*	1,000	2	*β*_1_x_1_ *+ β*_2_x_2_ *+ β*_3_x_1_ *◦* x_2_* + u + **ϵ***	*c_1_* = c_2_ = 0.05, *c*_3_ = 0.2	*𝒩 * (0, σ^2^)
*F*	1,000	2	*β*_1_x_1_ *+ β*_2_x_2_ *+ β*_3_x_1_ *◦* x_2_* + u + **ϵ***	*c*_1_ = c_2_ = 0.01, c_3_ = 0.28	*𝒩 * (0, σ^2^)
*G*	1,000	1	*β*_1_x_1_ *+ u + **ϵ***	*c*_1_ = 0.3	Γ(0, σ^2^/4)
*H*	1,000	1	*β*_1_x_1_ *+ u + **ϵ***	*c*_1_ = 0.3	Γ(0, σ^2^)
*I*	1,000	5	$\Sigma_{i=1}^{5}$ *β*_i_x_i_ + u + ***ϵ***	c_i_ ∼ *𝒩 *(6, 2^2^)/100, i = 1, . . . , 5	*𝒩 * (0, σ^2^)
*J*	1,000	20	$\Sigma_{i=1}^{20}$ *β*_i_x_i_ + u + ***ϵ***	c_i_ ∼ *𝒩 *(1.5, 0.5^2^)/100, i = 1, . . . , 20	*𝒩 * (0, σ^2^)
*K*	1,000	50	$\Sigma_{i=1}^{50}$ *β*_i_x_i_ + u + ***ϵ***	c_i_ ∼ *𝒩 *(0.6, 0.2^2^)/100, i = 1, . . . , 50	*𝒩 * (0, σ^2^)
*L*	1,000	100	$\Sigma_{i=1}^{100}$ *β*_i_x_i_ + u + ***ϵ***	*c_i_ ∼ *𝒩 ** (0.3, 0.1^2^)/100, i = 1, . . . , 100	*𝒩 * (0, σ^2^)

The first column Sim indicates the simulation setting, *n* is the number of samples, *k* the number of causal markers, y the formula of the phenotype, *c* the effect size of the causal markers, and ***ϵ*** the added noise. Here, x*_i_* denotes a single marker and x*_i_* ◦ x*_j_* is the Hadamard product of two markers.

### 2.3 Phenotype prediction models

Since literature does not show a predominant phenotype prediction model (see *Introduction*), we included 12 different prediction models in our comparative study. In the following, we first describe classical, statistical prediction models. Afterward, machine learning- and deep learning-based approaches are outlined. 

#### 2.3.1 Statistical prediction models

As baseline comparison partner, we included RR-BLUP (Meuwissen et al., 2001). Given *n* individuals and *m* markers, RR-BLUP is based on the following linear mixed model:


(4) y=μ1+Xw+ϵ,

where y *∈ ℝ^n^* denotes the vector of phenotypic observations,  1 *∈ * ℝ*^n^* denotes a vector of ones, and *μ ∈* ℝ is the overall mean. Further, ***X** ∈ ℝ^n*×*m^* is the genotype matrix, w *∈ ℝ^m^* contains the corresponding marker effects which are assumed to follow a Gaussian distribution with zero mean and a variance of *σ* ^2^ *_g_ **I***, with identity matrix ***I** ∈ ℝ^n*×*n^*, and ***ϵ** ∈* ℝ^*n*^ denotes the vector of residuals, with ***ϵ*** ∼ *𝒩* (0, *σ* ^2^ *_e_ ***I****).

Bayesian linear regression models are based on the same linear mixed model as RR-BLUP (see Equation 4). However, here the variance *σ* ^2^ *_e_* of the residuals is commonly assigned a scaled-inverse chi-squared distribution and the prior distribution of the marker effects w differs for each Bayesian model (Gianola, 2013). For instance, the prior distribution of w in case of Bayes A is a scaled *t*-distribution (Meuwissen et al., 2001). On the other hand, the priors of the models Bayes B and Bayes C both consist of a mixture of two distributions: one with a point of mass at 0 and 1 with a large variance. For Bayes B, this is a scaled *t*-distribution (Meuwissen et al., 2001), and Bayes C uses a normal distribution (Habier et al., 2011).

#### 2.3.2 Machine learning methods

In addition to RR-BLUP and Bayesian models, we included regularized linear regression models, namely, the Least Absolute Shrinkage and Selection Operator (LASSO) (Tibshirani, 1996) and Elastic Net (Zou and Hastie, 2005). For both models, the weights w can be estimated *via*


(5) argminw 12∥y −X*w ∥22+αΩ(w ),

where ***w***= (*w*_0_,*w*_1_,.., **w*_m_*)*^T^ ∈ ℝ ^m^* ^+1^ contains the bias term **w**_0_ *∈ ℝ* as well as the model’s coefficients ***w**_k_ ∈ ℝ* and ***X*** ^*^ *∈ ℝ ^n*×*m^* ^+1^ contains a vector of ones and the genotype matrix ***X** ∈ ℝ ^n*×*m^*. For regularization, both models employ a penalty term Ω(w) weighted by *α ∈ ℝ*_>0_. LASSO adds a sparsity constraint, which means that the absolute value of the weights (L1-norm) is used for penalization, i.e., Ω(w) = ∥w∥_1_. This is often considered as an automatic feature selection, as the weights of unimportant features are pushed toward zero. Elastic Net employs a weighted sum of the L1- and L2-norm (quadratic term penalizing the weights’ size), i.e., Ω(w) = λ∥*w*∥^2^_2_ + (1-λ)∥*w*∥_1_. As a consequence, Elastic Net combines the automatic feature selection effect of LASSO as well as a distribution of the influence among correlated features due to the L2-norm (James et al., 2017). While the hyperparameter *α* controls the strength of the regularization term Ω(w), λ gives the ratio between L1- and L2-regularization in Elastic Net.

Further, we included Support Vector Regression (SVR) as a kernel-based approach (Smola and Schölkopf, 2004). SVR allows the usage of non-linear kernel functions, *via* the so-called kernel-trick, enabling to find solutions for non-linear problems in an implicit higher-dimensional space. The aim is to construct a function that is at most a certain threshold away from the target values of the training data, while at the same time penalizing too complex models. Specifically, a hyperplane with the goal of maximizing the number of samples within a certain decision boundary is fitted. This is achieved by minimizing the term


(6) $$\min\;\frac{1}{2}∥w{∥}_{2}^{2}+C\sum_{i=1}^{n}\left(\xi_{i}+\xi_{i}^{*}\right)$$

(7) subject to{y i−w Tx [i]−w 0≤ϵ+ξiw Tx [i]+w 0−y i≤ϵ+ξi*ξi,ξi*≥0

where ***x*** ^[i]^ *∈ ℝ ^m^* is the genotype vector of the *i^th^* sample, w_0_ *∈ ℝ* denotes the bias, w *∈ ℝ ^m^* denotes the parameters of the SVR model, $\xi_{i},\xi_{i}^{*}\in \mathbb{R}$ are so-called slack variables, and *ϵ ∈ ℝ* determines the allowed deviation for any sample from the true targets *y_i_* (all slack variables for samples within the decision boundary are set to zero). *C ∈ ℝ* denotes the strength of the penalization due to deviations from the decision boundary and is usually optimized during hyperparameter tuning. Smaller *C* values focus more on the minimization of the L2-norm of the model parameters and thus may lead to a smoother and flatter decision function while allowing more prediction errors. By contrast, if *C* is larger, more emphasis is put on the penalization of model errors, which is more prone to overfitting (Drucker et al., 1997; Smola and Schölkopf, 2004).

Beyond that, ensemble learners, such as Random Forest (RF) (Breiman, 2001), showed a good performance in several phenotype prediction studies (Liu et al., 2019). For RF, an ensemble is constructed with Decision Trees, based on a random subsample of the training data. In the case of a regression task, the final prediction value is the mean over all these weak learners, which shall prevent overfitting. Similarly, XGBoost (XGB) (Chen and Guestrin, 2016) consists of multiple Decision Trees but employs gradient boosting. This means that weak learners are added sequentially, guided by the reduction in the gradient of the loss function to specifically focus on weaknesses of the current ensemble. With respect to the bias-variance trade-off, RF’s bagging strategy reduces variance, whereas boosting employed by XGB aims to reduce bias (James et al., 2017).

#### 2.3.3 Deep learning approaches

Eventually, we included three different types of neural networks, i.e., a feedforward Multilayer Perceptron (MLP), a Convolutional Neural Network (CNN), and a Local Convolutional Neural Network (LCNN). We designed the architecture of the MLP with different building blocks, consisting of fully connected, batch normalization and dropout layers. The number of these blocks and the number of neurons in each of the fully connected layers were considered as hyperparameters for optimization. After these blocks, a fully connected output layer is added, which delivers the final prediction (Srivastava et al., 2014; Ioffe and Szegedy, 2015; Goodfellow et al., 2016). For CNN and LCNN, the SNP matrix is encoded differently compared to the other methods, using a one-hot encoding (see *Experimental settings*). The CNN is composed of an optimized number of blocks, which consists of a convolutional layer, followed by a batch normalization and a dropout layer. After these blocks, max pooling is used for downsampling prior to a flattening layer. The prediction value is determined by a fully connected output layer with a preceding block of another fully connected, a batch normalization and a dropout layer (Srivastava et al., 2014; Ioffe and Szegedy, 2015; Goodfellow et al., 2016). Important parameters such as the kernel size and the stride are optimized. From a theoretical perspective, LCNNs provide an interesting property for phenotype prediction as they consist of region-specific filters. This addresses that certain sequences of markers can have totally different effects in different genome regions (Pook et al., 2020). Locally connected layers quickly lead to a large number of parameters. Consequently, we designed the architecture of the LCNN with a locally connected layer, followed by batch normalization, dropout, and max pooling layer. The output of this network part is flattened and further processed by an optimized number of blocks of fully connected, batch normalization and dropout layers, prior to a fully connected output layer. Due to the large amount of input features in relation to the sample size, regularization techniques are essential for phenotype prediction tasks. Besides the abovementioned dropout layers, we applied early stopping. Thereby, the loss on a validation set (independent data) is monitored during training, and if there is no improvement for a certain period, the optimization gets terminated (Bergstra et al., 2011). Preliminary experiments with an additional L1-regularization, penalizing the size of the neural network weights, did not show an improvement. For the training of the neural networks, we applied the Adam optimizer (Kingma and Ba, 2015). A rectified linear unit (ReLU) and a hyperbolic tangent were used as the non-linear activation function, with the selection depending on the hyperparameter optimization.

#### 2.3.4 Hyperparameter optimization

For hyperparameter search, we applied Bayesian Optimization using the framework Optuna (Akiba et al., 2019). In contrast to common optimization techniques, such as Random Search (Bergstra and Bengio, 2012), Bayesian Optimization tries to guide the search into promising directions using knowledge of already tested parameter settings. For that purpose, an objective value needs to be defined, in our case the performance on the validation data. Based on this, a probabilistic model that maps certain parameter combinations to a probability of a score on the objective function can be formulated. This enables the determination of promising parameter settings for further trials. In summary, Bayesian Optimization is computationally more expensive regarding the determination of candidate parameters but potentially more efficient as hyperparameters are suggested considering prior performances. For efficiency reasons, we also included a pruning strategy that stops trials if the intermediate result is worse than the 80*^th^* percentile of previous trials at the same step. For all prediction methods, we ran 200 trials each. In **Supplementary Tables S2** and **S3**, we show an overview of the hyperparameters and their ranges for each prediction model.

### 2.4 Experimental settings

After the data was prepared as described in *Synthetic data* and *Real-world data*, we encoded the SNP data using an additive genotype encoding, i.e., 0 for the homozygous major allele, 1 for the heterozygous allele, and 2 for the homozygous minor allele (for RR-BLUP -1/0/1, respectively). For CNN and LCNN, we used an alternative one-hot encoding. For instance, the nucleotide sequence ACGT is encoded as follows with a one-hot encoding: A → [1,0,0,0], C → [0,1,0,0], G → [0,0,1,0], T → [0,0,0,1]. The one-hot encoding allows to preserve the whole nucleotide information from the input data and might thus be more informative than the commonly used additive encoding. This encoding can be easily handled by a CNN and an LCNN.

To obtain an empirical estimate for different data splits, we performed a nested cross-validation with three outer folds and five inner folds. In order to guarantee that the phenotypic distribution stays approximately the same across all data splits, we first grouped the samples into bins with respect to the phenotypic values and used stratified splits afterward. We chose a nested cross-validation for performance estimates that are less biased by the random selection of the test samples. The mean performance on the inner folds was used as an objective value for the Bayesian hyperparameter optimization, with a potential pruning based on the intermediate result on each of them. Finally, the prediction model was retrained on the whole training and validation data using the best hyperparameters found in the 5-fold inner cross-validation. Then, each model’s performance was estimated using the hold-out test data. All optimizations were performed in Python 3.8 using the phenotype prediction framework easyPheno, which is publicly available at https://github.com/grimmlab/easyPheno. Bayesian methods are included using the R package BGLR (Pérez and de los Campos, 2014).

As evaluation metric, we determined the variance of the target variable y that can be explained with the prediction values $\hat{y}$ . Thus, the explained variance *v* is defined as follows:


(8) $$\nu \left(y,\hat{y}\right)=1-\frac{Var\left(y-\hat{y}\right)}{Var\left(y\right)}$$

Due to the nested cross-validation, we calculated the average and standard deviation of the test results on the three outer folds for an estimate of the model performance in the simulated circumstances.

### 2.5 Evaluation of selected features

In addition, we evaluated the importance of the features selected by the statistical prediction models RR-BLUP and Bayes B, the regularized regression approaches LASSO and Elastic Net, and the ensemble learners RF and XGB. To evaluate which markers were considered predictive by the models, we used the learned coefficients of LASSO, Elastic Net, RR-BLUP, and Bayes B as well as the feature importance of RF and XGB. For RF and XGB, we considered the normalized reduction in the optimization criterion by a certain feature. This reduction is averaged over all weak learners where the feature is used in the ensemble (Breiman, 2001). We then ranked the markers based on their feature importance and selected the top 1,000. For a better comparison between the results of the different models, we applied a min–max normalization. With regard to nested cross-validation, we assigned a feature importance averaged over all outer folds to each marker that was among the top 1,000 in at least one fold. If this averaged feature importance differs from zero, we subsequently consider the related feature as an important feature for the specific prediction model. However, for some prediction models, it is difficult to set a feature importance exactly to zero. To address this issue, we additionally filtered the abovementioned important features for those whose absolute values are at least 1% of the largest feature importance of that model.

For the synthetic phenotypes, we compared the importance of the selected features with the simulated effect sizes *β* and *γ*. With this, we can assess if the models captured the underlying structure of the simulated phenotypes by evaluating whether the causal and background SNPs were considered as important features with a similar magnitude by a prediction model. Since we do not know the ground truth regarding important SNPs for the real-world data, we performed genome-wide association studies (GWAS) to estimate which of the markers are statistically associated with the trait of interest and compared the selected features with the GWAS results. For this purpose, we used permGWAS (John et al., 2022), a permutation-based linear mixed model for phenotypes with normal or skewed distributions, and EMMAX (Kang et al., 2010). In contrast to the phenotype prediction models used in this study, in GWAS one only performs univariate statistical tests to test whether a single marker is associated with a certain phenotype, while at the same time correcting for population structure. Hence, for each SNP we obtain a p-value, where a smaller value indicates a stronger association. For all *Arabidopsis thaliana* traits, we checked the feature importance of the selected markers against the top 1,000 GWAS results (i.e., the 1,000 markers with the lowest p-values). For the corn and soy traits, we compared the feature importance of the algorithms with the top 100 GWAS results, since both datasets contain less than 10,000 markers.

## 3 Results and discussion

In the following section, we first summarize and discuss the results on synthetic data, including analyses of the selected features. Next, we provide an overview of the real-world results and discuss them and compare GWAS results with the selected features of some of the prediction methods. As is observed in **Supplementary Tables S4** and **S5**, both our experiments on synthetic and real-world data only showed minor differences between the methods from the Bayesian alphabet with Bayes B slightly outperforming the others. Hence, we subsequently focus on Bayes B, where we used 6,000 iterations and a burn-in of 1,000 for the training. Detailed results of the whole hyperparameter optimizations for all models and phenotypes can be found in our GitHub repository: https://github.com/grimmlab/phenotype_prediction.

### 3.1 Synthetic data

As described in *Materials and methods*, we conducted several experiments on synthetic data to analyze the behavior of phenotype prediction models under predefined settings. A detailed description of the synthetic data generation and all simulation configurations can be found in *Synthetic data in Materials and methods*.

#### 3.1.1 Prediction results

**Figure 1:**
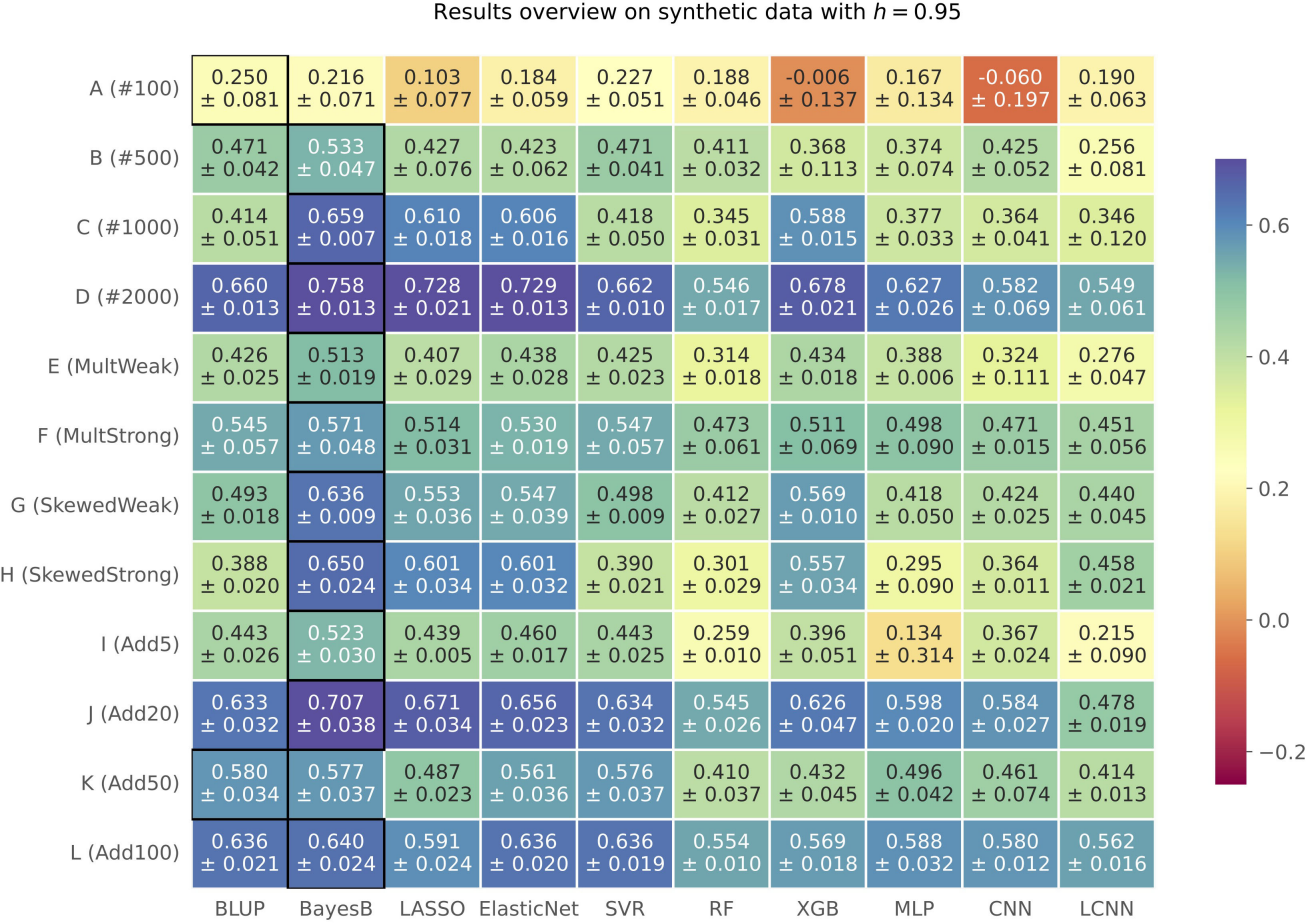
Results on synthetic data with *h*=0.95 shown in a heatmap: Each cell gives the explained variance *v* that the prediction model given on the horizontal axis achieved for the simulation configuration specified on the vertical axis. The color of each cell ranging from dark red to dark blue represents the prediction performance. The best result for each simulated phenotype is highlighted by a black frame around the cell.

We compared the results of 10 phenotype prediction methods on synthetically generated phenotypes for three different heritabilities *h ∈* {0.7, 0.85, 0.95}. Explained variance estimates *v* for all prediction methods and simulation settings are summarized in a heatmap (see **Figure 1** for *h*=0.95 and **Supplementary Figures S2** and **S3** for *h*=0.7 and *h*=0.85, respectively) Across all the simulated heritability settings, Bayes B showed the best performance in 29 out of 36 simulations (for *h*=0.7 in 9 out of 12, for *h*=0.85 in 10 out of 12, and for *h*=0.95 in 10 out of 12). RR-BLUP was the best-performing method in three cases (once for *h=*0.85 and twice for *h*=0.95), XGB on two phenotypes for *h*=0.7, and LASSO as well as SVR for one trait each, for *h*=0.7 and *h*=0.85, respectively. The top performer Bayes B is often closely followed by LASSO, Elastic Net, SVR, and XGB across all three heritabilities. Furthermore, RR-BLUP showed a good overall performance in several cases. Interestingly, none of the three neural network-based approaches wins in any of the 36 simulation settings. Furthermore, when comparing the results of these three prediction models to each other, we cannot determine a clear winner but slight advantages for the MLP with a good performance in general for some traits.

Overall, the results show an improvement with an increasing heritability *h*, which represents a stronger relationship among samples and less influence of random noise in case of our synthetic data generation model. In the following, we will discuss the results for a heritability of *h*=0.95 in more detail. Results for *h*=0.7 and *h*=0.85 can be found in the Supplementary (**Supplementary Figures S2**, **S3**). First, we analyzed the effect of increasing sample sizes, i.e., simulation settings *A* (#100) to *D* (#2000). The results show in general an improvement of the prediction results with an increasing number of samples across all heritabilities. However, we also observed a drop in performance for the simulation setting C (#1000) with *h*=0.95 for four out of 10 prediction models. One hypothesis in plant phenotype prediction is that neural network methods have great potential and might outperform other approaches with a higher number of samples being available (Montesinos-López et al., 2018b; Pook et al., 2020; Montesinos-López et al., 2021). However, we cannot confirm this in our simulations with increasing sample sizes (*A* (#100) to *D* (#2000)), since a deep learning-based method is never best overall (see **Figure 1**). In general, we can see that neural networks perform better with increasing sample sizes, but the same is true for other prediction methods. This suggests that the phenotype prediction is at least not only impeded by the comparably low amount of available data but also by the underlying genomic architecture.

We simulated weak (*E*) and strong multiplicative (*F*) effects. These differ regarding the effect size of the multiplicative term of the simulated causal SNPs, which is 0.2 for *E* and 0.28 for *F*. In **Figure 1**, we observe an improvement of the results for the scenario with the stronger multiplicative effect. This does not apply for *h*=0.7 and *h*=0.85. Across all three heritabilities, the results of the neural network-based methods improved relative to the best-performing approaches for strong multiplicative effects (setting *F*). This suggests that neural networks might be a valid option in case of highly non-linear phenotypic architectures. For all six scenarios with a skewed distribution of the simulated phenotype (*G* (SkewedWeak) and *H* (SkewedStrong)), Bayes B showed the best performance. For the stronger skewed scenario *H* with *h*=0.95, the performance of the MLP and CNN drops, while this does not apply for the LCNN.

Considering traits with multiple additive causal SNPs (*I* (Add5) to *L* (Add100)) across all three heritabilities, we observe that Bayes B performs best, mainly followed by RR-BLUP, LASSO, Elastic Net, and SVR with a similar performance and outperformed once by the former. If also considering configuration *C* (#1000) with similar settings but one causal SNP, the advantage of Bayes B tends to decrease with a growing number of causal SNPs. The formulation of Bayes B usually forces unimportant weights toward zero (but potentially not exactly to zero). This effect seems to be more profitable for scenarios with less causal SNPs for which the effect sizes of the causal SNPs also differ more from the background. Consequently, Bayes B can better capture the lower number of causal SNPs with larger effect sizes for scenarios *C* (#1000) and *I* (Add5), while this advantage is smaller for scenarios *J* (Add20), *K* (Add50), and *L*(Add100) with more causal markers that have smaller effect sizes. Beyond that, the performance of the MLP and the CNN is rather comparable to the top result for simulation configurations with multiple additive markers. Less causal SNPs can be considered as more noise in the features, which is hard to deal with for the neural network-based approaches if also considering the relatively low number of samples.

In summary, the results of the synthetic data suggest a better performance for less complex methods, such as Bayes B. More advanced approaches, such as deep learning-based models, do not seem to improve the predictions, which is in accordance with existing literature (Ma et al., 2018; Azodi et al., 2019; Crossa et al., 2019; Pook et al., 2020). In most cases, classical ML-based techniques, such as Elastic Net, SVR, and XGB, show a comparable or even the best outcome. The good performance of Bayes B, RR-BLUP, and the classical ML-based approaches might be explained in parts by the smaller number of hyperparameters that need to be optimized in comparison with the neural network-based models, having the relatively low number of samples in mind. With Bayesian Optimization, we applied a state-of-the-art hyperparameter search. However, as all prediction models were optimized using the same number of trials, we might still get a better hyperparameter setting for less complex approaches.

A further reason for the success of Bayes B, LASSO, and Elastic Net might be the inbuilt feature selection. This is especially beneficial in phenotype prediction settings because of the large amount of features in comparison with available samples. Nevertheless, L1-regularization did not show an improvement for preliminary experiments on neural network-based approaches.

#### 3.1.2 Feature importance analysis

To evaluate how well the different models are able to capture the determinant markers of synthetic phenotypes, we compared the effect sizes *β* and *γ *with the feature importances of RR-BLUP, Bayes B, LASSO, Elastic Net, RF, and XGB, as described in *Evaluation of selected features*. For the subsequent analysis, we focused on Bayes B, Elastic Net, and XGB as the top performers of the statistical prediction models, regularized linear regression approaches, and ensemble learners, respectively. In **Table 2**, we summarize the results for a heritability of *h*=0.95. Similar statistics for all three heritabilites and all six prediction models can be found in **Supplementary Tables S6–S8**. In these Supplementary Tables, we show both all selected features with a weight not equal to zero and results when filtering out all features that are less than 1% of the largest feature importance. Across all three heritabilities, we observed in almost every simulation setting that LASSO selects the least number of features. This is not surprising due to the sparsity constraint of the LASSO model. Furthermore, besides scenarios with few samples (*A* (#100) and *B* (#500)) and multiple additive markers (*K* (Add50) and *L* (Add100)), Elastic Net tends to select the second least features, indicating a strong effect of the L1-regularization part. Without filtering out feature importances smaller than 1% of the largest feature importance, Bayes B selects many features, similar to RR-BLUP, RF, and XGB. However, when considering this filter, the number of important features for Bayes B decreases significantly in many simulation settings (see **Supplementary Tables S6-S8**). This effect tends to become weaker or even to vanish with a larger number of samples (*I* (Add5) to *L* (Add100)) and for scenarios with a stronger multiplicative effect (*F* (MultStrong)). Furthermore, it cannot be observed for scenario *A* (#100) with only 100 samples. We see a similar behavior for the other prediction methods, but not as strong as for Bayes B.

**Table 2:** Analysis of feature importances of Elastic Net, Bayes B, and XGB for synthetic data with *h*=0.95.

Sim	Important features			Background SNPs			Causal SNPs		
	ElasticNet	BayesB	XGB	ElasticNet	BayesB	XGB	ElasticNet	BayesB	XGB
A (#100)	733	2327	87	90 (12%)	265 (11%)	11 (13%)	1/1 [2]	1/1 [1]	1/1 [49]
B (#500)	1466	2124	890	152 (10%)	243 (11%)	105 (12%)	1/1 [1]	1/1 [1]	1/1 [1]
C (#1000)	541	2134	1749	94 (17%)	274 (13%)	232 (13%)	1/1 [1]	1/1 [1]	1/1 [3]
D (#2000)	1004	2112	2479	186 (19%)	322 (15%)	357 (14%)	1/1 [1]	1/1 [1]	1/1 [4]
E (MultWeak)	1639	2080	1928	220 (13%)	260 (12%)	236 (12%)	2/2 [1,2]	2/2 [1,2]	2/2 [9,18]
F (MultStrong)	1595	2094	1897	192 (12%)	260 (12%)	221 (12%)	2/2 [1,134]	2/2 [1,57]	2/2 [30,250]
G (SkewedWeak)	441	2072	2528	82 (19%)	258 (12%)	316 (12%)	1/1 [1]	1/1 [1]	1/1 [3]
H (SkewedStrong)	387	2028	1993	64 (17%)	217 (11%)	259 (13%)	1/1 [1]	1/1 [1]	1/1 [2]
I (Add5)	1775	2105	2590	237 (13%)	268 (13%)	333 (13%)	5/5 [1,3,6,10,17]	5/5 [2,4,6,7,10]	5/5 [5,66,328,693,2195]
J (Add20)	1181	2217	1593	147 (12%)	262 (12%)	213 (13%)	20/20 [12 in top20]	20/20 [13 in top20]	19/20 [2 in top20]
K (Add50)	2122	2081	1991	263 (12%)	253 (12%)	244 (12%)	46/50 [15 in top50]	45/50 [15 in top50]	28/50 [0 in top50]
L (Add100)	2000	2091	1824	244 (12%)	260 (12%)	231 (13%)	70/100 [26 in top100]	67/100 [27 in top100]	43/100 [1 in top100]

For each simulation configuration and model, the table shows the number of SNPs deemed as an important feature for at least one of the outer folds in the nested cross-validation. Furthermore, the number of background SNPs within the important features is stated as well as the ratio between the found background SNPs and the total amount of important features as percentage value in parentheses, i.e., the true positive rate (TPR). For the causal SNPs, we show the number of causal SNPs deemed important by each algorithm and in brackets the ranking of the causal SNPs within the important features. For configurations *J*, *K* and *L*, we give the number of causal SNPs within the k (total number of causal SNPs) most important features.

Further, we analyzed how many of the actual background SNPs were identified by each prediction model. We can observe that both Bayes B and XGB usually recognized more of them. However, setting the number of detected background SNPs in relation to the number of selected features, which we subsequently call the true positive rate (TPR) and which is given in parentheses in **Table 2**, Elastic Net shows the best performance for seven of the 12 scenarios. With respect to all heritabilities and prediction models (see **Supplementary Tables S6-S8**), LASSO mostly selected a larger amount of actual background markers in relation to the total number of important features in comparison with the other prediction models, probably due to L1-regularization. Again, when removing very small feature importances, the observation regarding the TPR changes heavily (see **Supplementary Tables S6-S8**). For most simulation settings, the TPR of Bayes B increases significantly, generally outperforming the other prediction methods. Again, as the filtering effect for determining the important features is weaker with a growing number of multiple causal markers (*I* (Add5) to *L* (Add100)), stronger multiplicative effects (*F* (MultStrong)), and a small number of samples (*A* (#100)), the increase in the TPR is also less pronounced or not existing.

Without considering scenarios with multiple additive effects (*I* (Add5) to *L* (Add100)), the simulated causal SNPs were identified by all prediction models except RR-BLUP in all 24 scenarios (**Supplementary Tables S6-S8**). With respect to scenarios *I* (Add5) to *L* (Add100), the number of detected causal SNPs decreased with a growing amount of additive markers. This is expected, as the individual effect sizes also decreased with a higher number of causal markers. Consequently, they were harder to distinguish from background or non-related SNPs. In general, Elastic Net and Bayes B ranked the causal SNPs higher than XGB for these scenarios. Considering all prediction models and heritabilities (**Supplementary Tables S6-S8**), we observe that RF also ranks the causal SNPs similar to XGB, whereas they are more important for LASSO, Elastic Net, and Bayes B.

In summary, LASSO, Elastic Net, and Bayes B seem to perform the best feature selection with respect to our simulations. The good feature selection of LASSO and Elastic Net is probably caused by the L1-regularization term, which forces unimportant features to zero. When filtering out very small feature importances, Bayes B shows the best feature selection. Consequently, it is able to identify the predictive markers but cannot set the weights of less important SNPs to exactly zero. However, their weights are rather small (less than 1% of the largest coefficient), so their influence is limited. Probably, this good feature selection is also a reason for the good prediction performance of LASSO, Elastic Net, and Bayes B.

To further analyze the actual values of the feature importances and not only if an SNP was selected at all, we show the min–max normalized feature importances (without filtering) of Bayes B, Elastic Net, and XGB in comparison with the effect sizes for *h*=0.95 in **Figure 2**. Similar plots for the other heritabilities as well as plots showing only one of the prediction models and consequently without overlap can be found in the Supplementary (**Supplementary Figures S4-S23**).

**Figure 2:**
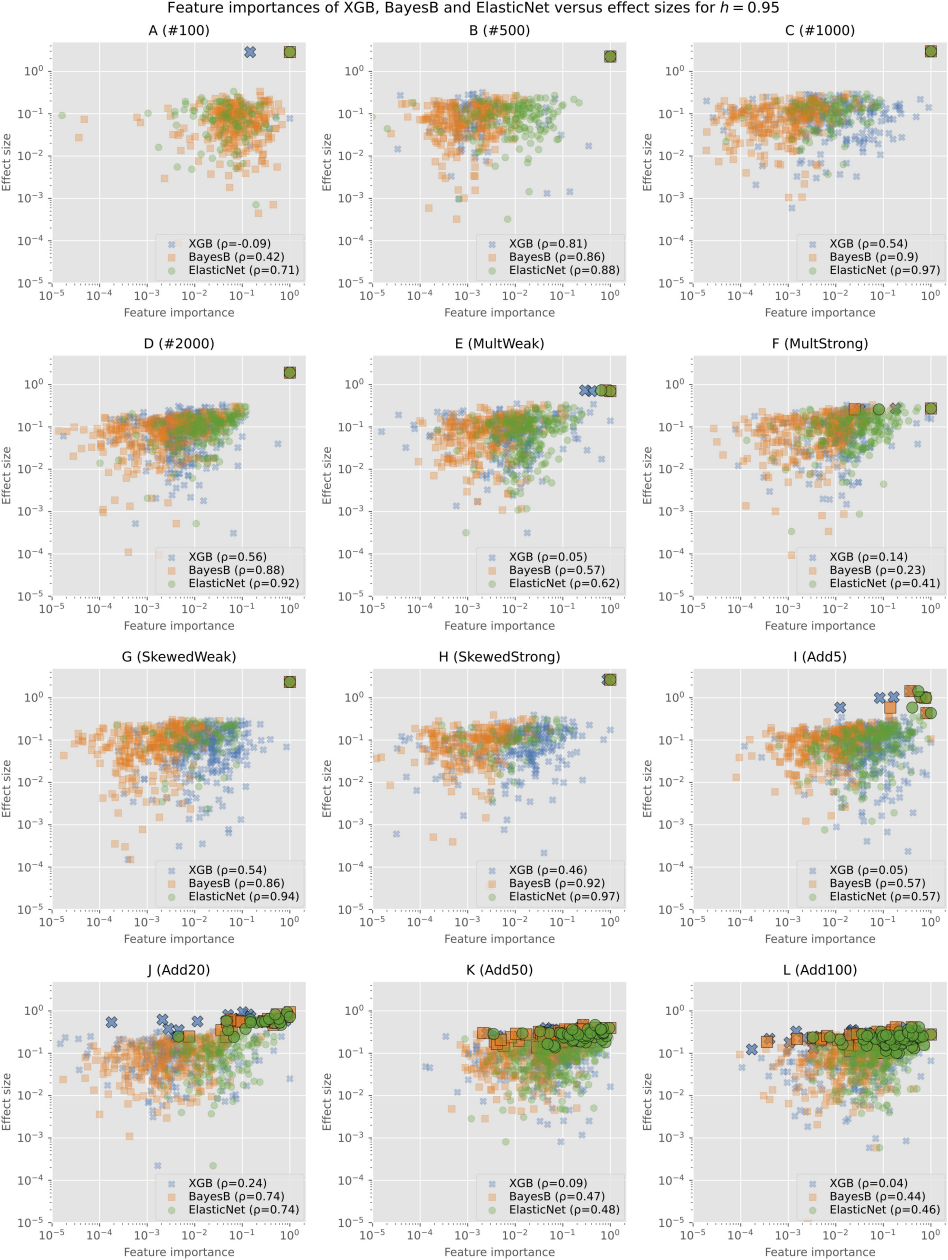
Min–max normalized feature importances of Bayes B, Elastic Net, and XGB in comparison with effect sizes on synthetic data for *h*=0.95: Each subplot shows the results of one of the simulation configurations on a logarithmic scale. Only SNPs for which both the effect size and the feature importance are not zero are shown. Causal SNPs are highlighted by a larger marker size and a black frame. The legend additionally gives the Pearson correlation coefficient of the effect sizes and feature importances.

As Elastic Net rates less features as important, we see a smaller amount of points in the plots. The causal markers are highlighted by a larger marker size and with a black frame. In all plots, we can see that the causal markers are rated important by every algorithm. However, especially for XGB and in some cases for Elastic Net as well, we observe that background SNPs have often a similar feature importance (values close to maximum of 1 on the horizontal axis). As the effect sizes of the causal and background SNPs do not differ that much for scenarios with multiple causal markers (*I* (Add5) to *L* (Add100)), the distance between both types is also smaller in the plots. Further, we observe that feature importances of Bayes B and Elastic Net for the causal SNPs tend to be higher in comparison with XGB.

The majority of the background SNPs are similarly important for the prediction models as we observe large overlaps in all figures. In most cases, these clusters tend to be rather on the left and middle sides of the plot, indicating a bigger difference between the importance of the causal and background SNPs. For Bayes B and XGB, there are more markers that have a relatively high effect size but are rated unimportant (left upper and left middle parts of the plots). Further, XGB rates several SNPs with a low effect size important (right lower and right middle parts of the plots). Overall, in most scenarios, there are not many points in the lower range of the plots. This suggests that selected background SNPs are rather those with larger effect sizes.

In addition, we computed the Pearson correlation coefficient between the effect sizes and feature importances. In almost all cases, Elastic Net shows the highest correlation, closely followed by Bayes B or placed second after Bayes B. As our evaluations show that both Elastic Net and Bayes B perform a good selection of the important features, we assume that the - often slighly - better performance of Bayes B is caused by the selection of more background features, which apparently also contain information regarding the trait of interest.

### 3.2 Real-world data

Next, we compared the performance of the prediction models for several traits in real-world data, including the model organism *Arabidopsis thaliana* and two breeding datasets of corn and soy.

#### 3.2.1 Prediction results

**Figure 3:**
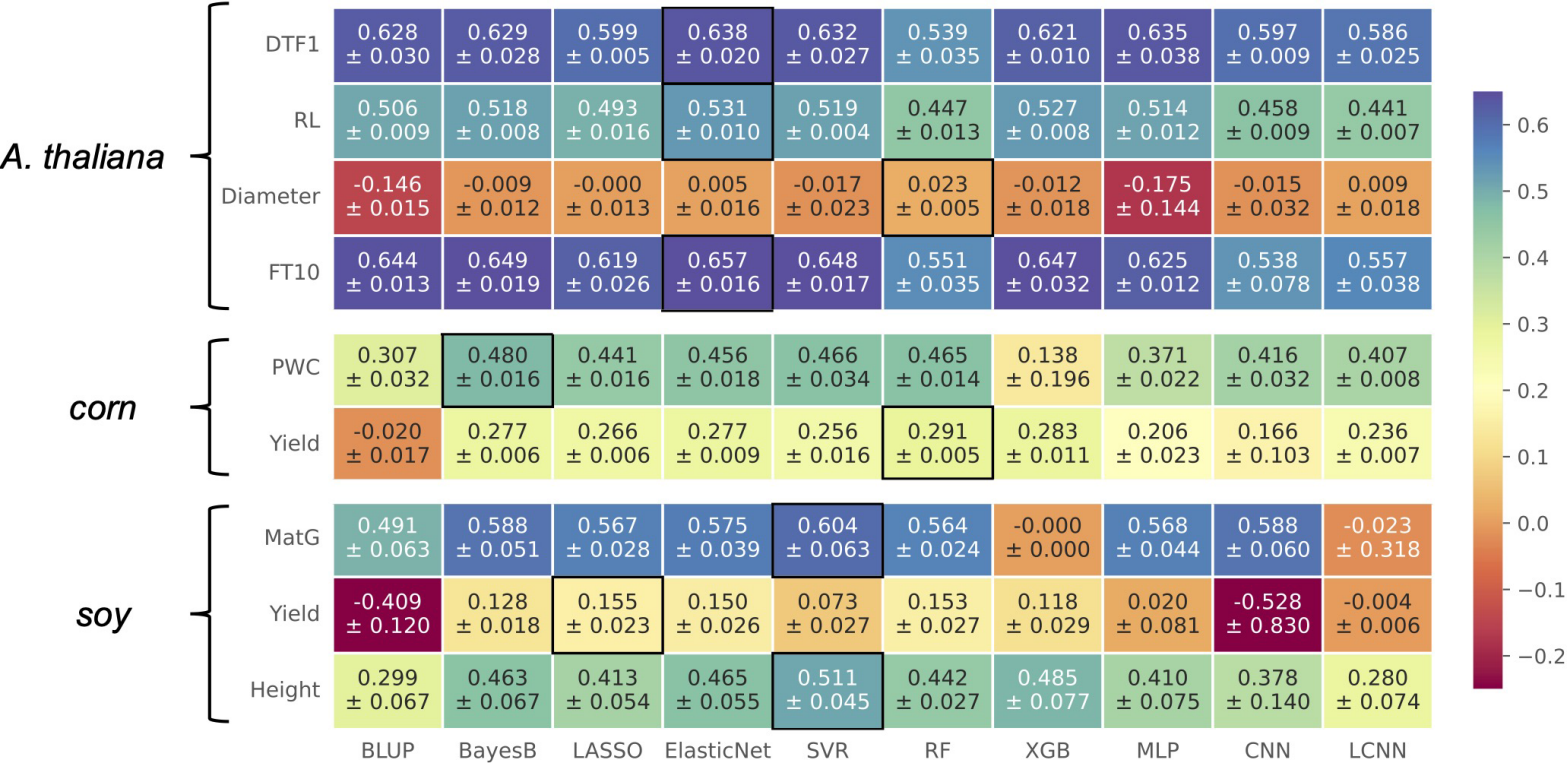
Results on real-world data for *A. thaliana*, soy, and corn shown in a heatmap: Each cell gives the explained variance *v* that the prediction model given on the horizontal axis achieved on the phenotype specified on the vertical axis. The color of each cell ranging from dark red to dark blue represents the prediction performance. The best result for each phenotype is highlighted by a black frame around the cell.

In general, no model performed best for all considered traits, as illustrated in the heatmap in **Figure 3**. This is in accordance with existing literature (Azodi et al., 2019). Especially for traits in *Arabidopsis thaliana*, we observe that multiple models deliver similar results with differences within the standard deviations. For corn and soy, this observation is not that clear. For three traits, Elastic Net is the top performer, often closely following the prediction models that led to the best results for the other phenotypes. For the traits Diameter in *Arabidopsis thaliana* as well as for Yield in soy, none of the models performed considerably well. The commonly used model Bayes B that performed best on synthetic data showed a competitive performance for most of the traits. Although LCNNs are supposed to better capture the influence of different marker effects than CNNs and MLPs, the LCNN was outperformed by at least one of the two other deep learning-based approaches for every phenotype except Diameter and corn’s Yield. Comparing the outcome of CNN and LCNN, which both used one-hot-encoded data, to the MLP, we observe that the MLP is superior in five cases. The MLP is furthermore competitive to the top performer for three traits in *Arabidopsis thaliana*. However, as the additive encoding still leads to a loss of information, this indicates that further encoding strategies should be investigated in the future.

If we recap the results on synthetic data, we observe that the best-performing prediction methods on real-world data also performed well. For instance, the top performer on real-world data, Elastic Net, showed its best performance for scenarios with strong multiplicative effects, skewed phenotype distributions, or multiple additive markers. Hence, an explanation for its good performance on real-world data might be that similar genotype–phenotype relationships are present.

Similar to the synthetic data, the real-world experiments do not reveal an advantage of neural network-based techniques over the other approaches with an increasing number of samples. Although all three neural networks improve with a growing sample size for the *Arabidopsis thaliana* traits, so do the classical ML approaches. Hence, the effect might rather be caused by the genetic architecture or the typical amount of available samples for plant phenotype prediction is still not enough.

#### 3.2.2 Feature importance analysis

To evaluate the feature importances of Bayes B, Elastic Net, and XGB for the real-world data, we first compared how many markers were found by each model and then checked these against GWAS results.

**Figure 4:**
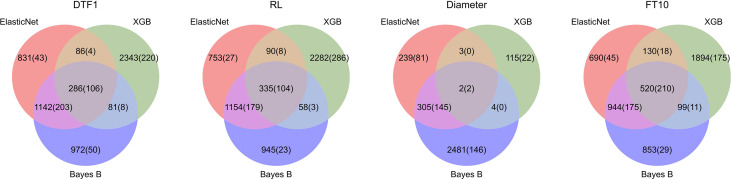
Comparison of feature importance of Elastic Net, Bayes B, and XGB with GWAS top 1,000 for *Arabidopsis thaliana*: For each phenotype, the corresponding Venn diagram shows the number of SNPs which were deemed important by one or more algorithms. A SNP is considered important if its related model parameter differs from zero in at least one of the outer folds in the nested cross-validation. The numbers of SNPs that were additionally within the top 1,000 GWAS results are shown in parentheses.

For *Arabidopsis thaliana*, we took the 1,000 most significantly associated markers, i.e., the 1,000 markers with the lowest p-values. **Figure 4** shows Venn diagrams for all *Arabidopsis thaliana* traits with the number of markers selected by each model (feature importance averaged over all outer folds differs from zero) and the corresponding number of top 1,000 GWAS results in parenthesis. Similar to the results on synthetic data, Elastic Net selects less features in comparison with Bayes B and XGB, probably due to the L1-regularization. For the *Arabidopsis thaliana* traits, we observe that the important SNPs overlap in many cases. For these intersections of all three prediction models, we see the highest ratios of SNPs also being within the top 1,000 GWAS results. Furthermore, Bayes B and Elastic Net agree on the importance of many features, which might be caused by similar underlying assumptions. On average, with a value of *2*2.5%, Elastic Net shows the highest ratio of selected features also being within the 1,000 considered GWAS results.

For soy and corn, around 600 and 7,000 SNPs were available, respectively. Hence, we only took the top 100 GWAS results for comparison. The results of the feature analysis for soy and corn can be seen in **Supplementary Figure S24**. As expected, less SNPs were considered important in general. For the corn trait PWC, we see that Bayes B found most of the GWAS results and also showed the best performance regarding the explained variance. However, Elastic Net only selects 12 top 100 GWAS results in comparison with 24 for Bayes B, but Elastic Net also performs comparably well for this phenotype. Further, XGB shows a low explained variance for MatG on soy, for which it selects none of the GWAS results, whereas the other two approaches select much more and achieve a much better performance (again with Bayes B selecting most of the top 100 GWAS results and the best performance among these three techniques). For Yield in soy, Bayes B selects most GWAS results but is outperformed by Elastic Net in terms of explained variance. For Height in soy, all three prediction models agree on most selected features and deliver a comparable explained variance. Interestingly, despite the preselection using QTL for soy, the ratios of top GWAS results within the selected features are smaller than for *Arabidopsis thaliana*.

Additionally, we compared the feature importances after removing those smaller than 1% of the largest one (see **Supplementary Figure S25**). However, similar to simulations *J* (Add20) to *L* (Add100), the difference to the feature importance without filtering is rather small. In general, there seems to be a connection between the GWAS results and feature importances. However, it is not clear to which extent this influences the predictive ability of a model.

## 4 Conclusion and future outlook

In this paper, we conducted a comparative study of 12 different phenotype prediction models and evaluated their performance on both synthetic and real-world data. Based on the synthetic data, we can conclude that Bayes B was the best-performing model, although it is a rather simple method. For more complex neural network-based techniques, we could not see an advantage within our simulations. With respect to real-world datasets, this observation is less clear. No prediction model performs best for all real-world traits, and many lead to comparable results. While Elastic Net outperformed all other comparison partners for three phenotypes, SVR and RF delivered the best result for two traits each. Both Bayes B and LASSO performed best in one case. Beyond that, a neural network-based approach never achieved the highest explained variance for the real-world traits but competitive results for some phenotypes.

Our results are in line with the findings of Azodi et al. (2019), as they also showed that depending on the species–trait combination, both linear and non-linear models perform equally well for phenotype prediction in terms of Pearson correlation coefficient. Contrary to our results on real-world data and in line with the experiments on simulated data, their best-performing model turns out to be a Bayesian approach. Furthermore, SVR is their best non-linear approach. In accordance with our study, deep learning-based approaches are never among the top performers. On the other hand, Pook et al. (2020) observed that LCNN has the highest average prediction ability compared to CNN and MLP in *A. thaliana* traits. However, we cannot confirm these results within our study. In accordance with our findings, they also showed that linear methods, e.g., Bayes B, outperformed all neural nets. Sandhu et al. (2020) showed that MLP and CNN both on average have an improved prediction performance compared to RR-BLUP in all five wheat traits and environments they considered. If comparing the explained variances for RR-BLUP with those of MLP and CNN in our study, we can observe similar results, although we do not consider additional environmental effects. MLP and CNN perform better or comparable to RR-BLUP in almost all nine traits. In general, all studies show that the prediction ability of the different models highly differs for the considered species and traits.

For both synthetic data as well as real-world data, we cannot observe a stronger relationship between neural network-based approaches and the number of available samples than we observe for the other prediction models. This suggests that the phenotype prediction capabilities are mainly influenced by the genetic architecture. For instance, Ubbens et al. (2021) formulate the theory that DL-based methods rather rely on genetic relatedness instead of single marker effects, probably due to so-called shortcut learning (Geirhos et al., 2020). Consequently, this still suggests a potential in neural network-based approaches if shortcut learning is overcome, even though sample sizes are rather small compared to other tasks, e.g., image processing. However, the performance of neural networks is highly influenced by their architectural design. Tasks based on genomic data might require the development of new neural network architectures. For instance, the number of weights that need to be optimized during training grows heavily with the input size. With SNP matrices consisting of hundreds of thousands or even millions of features, neural network training quickly becomes computationally exhaustive or even impossible, especially when considering the memory available on graphical processing units. Currently, it is common to reduce the amount of SNPs with methods such as LD pruning or dimensionality reduction approaches. However, this can cause a loss of information, which might lead to worse results on the prediction task. Hence, the design of neural network architectures that can handle larger SNP matrices is interesting for future research.

The corn and soy data used in this study are taken from commercial breeding programs. A breeding program aims to commercialize a new variety within a few years. It is thus usually highly focused on optimizing a population for the traits of interest. The genetic diversity is narrower compared to the *Arabidopsis thaliana* population. The relevant alleles for traits of interest in a commercial breeding program are also much more fixed, and SNP panels are preselected accordingly to save costs. This dependence of samples can be seen critical from an ML perspective, with a potential leak of information to test data. However, plant breeding companies rather aim to create a model that captures the genetics of their breeding program than one that generalizes well for unknown samples.

Although the corn and soy data used in this publication stem from the same environment, the whole breeding programs were actually conducted under several conditions. In addition to environmental effects, this also leads to different field management practices. Consequently, in contrast to *Arabidopsis thaliana*, which is typically grown under controlled environments (i.e., greenhouse or climate-controlled growth chambers), phenotypes in a commercial breeding program are typically influenced by environmental conditions impeding phenotype prediction. For this reason, the integration of environmental features to phenotype prediction models is highly relevant for future research. Several studies show advantages when integrating such additional information (Montesinos-López et al., 2018a; Sandhu et al., 2020). However, we focused solely on genotype information in this study, to get a better understanding of the genotype–phenotype relationship. In this context, it is challenging to control the influence of genomic data with probably way more features and the environmental factors. Furthermore, this leads to datasets with mixed input data types (e.g., continuous and discrete inputs) and even larger dimensionalities. Thus, research on representation learning for mixed data types to extract the relevant information has potential for future research. Beyond that, we focused on single-trait prediction in this study. As already mentioned in the *Introduction*, there are publications which show advantages of multi-trait prediction (Montesinos-López et al., 2018b; Sandhu et al., 2021). Hence, when further considering computational advantages of multi-trait prediction, this is an interesting direction for future research.

The analyses of the selected features in comparison with the known effect sizes on synthetic data showed advantages of the regularization in LASSO and Elastic Net. However, this was not observed in preliminary experiments with L1-regularization for neural network-based methods. Regularizing approaches such as dropout and early stopping apparently did not lead to competitive results. Nevertheless, as regularization is important for data with such a high dimensionality, the integration of further regularization measures could be essential for all other ML-based approaches as well.

Despite the fact that we considered various different simulated settings and three species, this study can only give first insights. Currently, it is not clear that our results generalize across further simulation designs and species. Additional experiments and research are required for a more general conclusion. As described above, this leads to many new research questions and a lot of potential for future research. Especially a further evaluation and design of neural network-based approaches specifically for phenotype prediction seem interesting.

## Data availability statement

The fully imputed SNP matrix data for *Arabidopsis thaliana* is publicly available and can be downloaded from https://doi.org/10.6084/m9.figshare.11346893.v1. Phenotypic data can be downloaded for free from the manually curated database AraPheno (https://arapheno.1001genomes.org). The following phenotypes have been used: FT10 (https://www.doi.org/10.21958/phenotype:261), DTF1 (https://www.doi.org/10.21958/phenotype:703), RL (https://www.doi.org/10.21958/phenotype:704), and Diameter (https://www.doi.org/10.21958/phenotype:707). All simulated phenotypes, detailed results of the whole hyperparameter optimization, precomputed permutation-based GWAS results, and the code for conducting the simulations and generating all figures can be freely downloaded from our GitHub repository: https://github.com/grimmlab/phenotype_prediction. 

## Author contributions

MJ, FH, and DGG contributed to conception and design of the study. PR, CM, and SJS provided the breeding datasets and performed the data preparation for the corn and soy datasets. RD performed the initial breeding data analysis and hybrid simulation. MJ prepared the *Arabidopsis thaliana* data and wrote the simulation scripts to generate synthetic phenotypes, with contributions from FH and DGG. FH and MJ implemented all prediction methods and performed all simulations and comparative and statistical analyses. MJ, FH, and DGG analyzed and interpreted the results. MJ, FH, and DGG wrote the first draft of the manuscript. RD, SJS, and CD contributed to sections of the manuscript. All authors contributed to manuscript revision and read and approved the submitted version.

## Funding

The project is supported by funds of the Federal Ministry of Education and Research (BMBF), Germany [01∣S21038].

## Acknowledgments

This article is funded by the Open Access Publication Fund of Weihenstephan-Triesdorf University of Applied Sciences. The authors gratefully acknowledge the compute resources provided by the Leibniz Supercomputing Centre (www.lrz.de). The authors are grateful for the permissions of data use granted by Computomics’ customers for the corn and soy datasets.

## Conflict of interest

Authors RD, CM, PR, CD, and SJS are/were employed by Computomics GmbH.

The remaining authors declare that the research was conducted in the absence of any commercial or financial relationships that could be construed as a potential conflict of interest.

The authors declare that this study received funding from the Federal Ministry of Education and Research (BMBF), Germany [01|S21038]. The funder was not involved in the study design, collection, analysis, interpretation of data, the writing of this article or the decision to submit it for publication.

## Publisher’s note

All claims expressed in this article are solely those of the authors and do not necessarily represent those of their affiliated organizations, or those of the publisher, the editors and the reviewers. Any product that may be evaluated in this article, or claim that may be made by its manufacturer, is not guaranteed or endorsed by the publisher.
