# Supplementary material for: A comparison of classical and machine learning-based phenotype prediction methods on simulated data and three plant species
Source: Front Plant Sci. 2022 Nov 4;13:932512. doi: 10.3389/fpls.2022.932512 (PMC9673477; doi:10.3389/fpls.2022.932512)
Supplement: Supplementary file 1 [file DataSheet_1.pdf]

frontiers

Supplementary Material

A comparison of classical and machine learning-based phenotype prediction methods on simulated data and three plant species

Maura John [1,2,†], Florian Haselbeck [1,2,†], Rupashree Dass [3], Christoph Malisi [3], Patrizia Ricca [3], Christian Dreischer [3], Sebastian J. Schultheiss [3] and Dominik G. Grimm [1,2,4,*]

[1] Technical University of Munich, Campus Straubing for Biotechnology and Sustainability, Bioinformatics, Straubing, Germany
[2] Weihenstephan-Triesdorf University of Applied Sciences, Straubing, Germany
[3] Computomics GmbH, Tübingen, Germany
[4] Technical University of Munich, Department of Informatics, Garching, Germany

[†]These authors have contributed equally to this work and share first authorship.
[*] Correspondence: Dominik G. Grimm, TUM Campus Straubing for Biotechnology and Sustainability & Weihenstephan-Triesdorf University of Applied Sciences, Petersgasse 18, 94315 Straubing, dominik.grimm@hswt.de

1 SUPPLEMENTARY TABLES

Table S1. Dataset statistics for all real-world phenotypes: For each real-world phenotype, the table shows the available number of samples and the number of SNPs after removing duplicates in additive as well as one-hot encoding. Further the overall mean, standard deviation, minimum and maximum are given.

	phenotype	#samples	#SNPs (additive)	#SNPs (one-hot)	mean	sd	min	max
A. thaliana	DTF1	936	44434	45205	64.49	26.13	23.5	139.0
	RL	850	43803	666436	42.17	16.85	7.50	99.0
	Diameter	656	44189	45193	15.32	3.25	6.00	24.00
	FT10	1058	45841	46366	83.46	17.88	50.75	157.50
corn	PWC	1797	7192	8373	20.89	0.90	17.50	24.80
	Yield	1411	7100	8350	234.19	22.70	130.50	299.20
soy	MatG	457	598	608	7.46	0.53	6.50	8.60
	Yield	456	598	608	72.64	5.25	53.51	84.61
	Height	460	598	608	89.52	14.54	59.57	131.28

Table S2. Hyperparameters and ranges optimized for LASSO, Elastic Net SVR, RF and XGB: Numbers in square brackets reflect a list of potential values or the lower respective upper bound, with a step size of 1 for integer values. In some cases, specific step sizes Δ were used.

Hyperparameter	Values	Notes
	LASSO	
alpha	$[10^{-3}, 10^{3}]$	weighting factor of the L1-regularization term
	ElasticNet	
alpha	$[10^{-3}, 10^{3}]$	weighting factor of the regularization terms
l1_ratio	$[0.05, 0.95]$ with $\Delta = 0.05$	trade off between L1- and L2-regularization
	SVR	
kernel	['linear', 'poly', 'rbf']	kernel function to use
C	$[10^{-3}, 10^{3}]$	regularization factor
degree	$[1, 5]$	polynomial degree of kernel function (*if kernel is 'poly'*)
gamma	$[10^{-3}, 10^{3}]$	kernel coefficient (*if kernel is 'rbf' or 'poly'*)
	RF	
n_estimators	$[50, 100, 250, 500, 750, 1000, 1250, 1500, 1750, 2000, 2250, 2500, 2750, 3000, 3500, 4000, 4500, 5000]$	number of trees in the ensemble
min_samples_split	$[0.005, 0.2]$ with $\Delta = 0.005$	minimum ratio of the number of samples to split a node
max_depth	$[2, 50]$ with $\Delta = 2$	maximum depth of a tree
min_samples_leaf	$[0.005, 0.2]$ with $\Delta = 0.005$	minimum ratio of the number of samples at a leaf node
max_features	['sqrt', 'log2']	number of features to consider at determining best split
	XGB	
n_estimators	$[50, 100, 250, 500, 750, 1000, 1250, 1500, 1750, 2000, 2250, 2500, 2750, 3000]$	number of trees in the ensemble
max_depth	$[2, 10]$ with $\Delta = 1$	maximum depth of a tree
learning_rate	$[0.025, 0.3]$ with $\Delta = 0.025$	boosting learning rate
gamma	$[0, 1000]$ with $\Delta = 10$	minimum loss reduction for a further partition on a leaf node
subsample	$[0.05, 0.8]$ with $\Delta = 0.05$	subsample ratio of training instances for tree construction
colsample_bytree	$[0.05, 0.8]$ with $\Delta = 0.05$	ratio of features to use for each tree
reg_alpha	$[0, 1000]$ with $\Delta = 10$	L1-regularization term on weights

Table S3. Hyperparameters and ranges optimized for MLP, CNN and LCNN: Numbers in square brackets reflect a list of potential values or the lower respective upper bound, with a step size of 1 for integer values. In some cases, specific step sizes Δ were used.

Hyperparameter	Values	Notes
	MLP	
dropout	$[0, 0.5]$ with $\Delta = 0.1$	dropout rate for dropout layers
act_function	['relu', 'tanh']	activation function to use
learning_rate	$[10^{-6}, 10^{-5}, 10^{-4}, 10^{-3}, 10^{-2}, 10^{-1}]$	learning rate of the Adam optimizer
early_stop_pat	$[0, 20]$ with $\Delta = 5$	epochs without improvement needed for early stopping
n_layers	$[1, 5]$	number of building blocks consisting of fully-connected, batch normalization and dropout layer
n_init_units_fac	$[0.1, 0.7]$ with $\Delta = 0.05$ if $n_{features} \leq 20.000$<br>$[0.1, 0.3]$ with $\Delta = 0.01$ if $n_{features} > 20.000$	number of neurons in the first fully-connected layer in relation to the number of input features
perc_dec	$[0.1, 0.5]$ with $\Delta = 0.05$ if $n_{features} \leq 20.000$	percentage decrease of number of neurons per building block
	CNN	
dropout	$[0, 0.5]$ with $\Delta = 0.1$	dropout rate for dropout layers
act_function	['relu', 'tanh']	activation function to use
learning_rate	$[10^{-6}, 10^{-5}, 10^{-4}, 10^{-3}, 10^{-2}, 10^{-1}]$	learning rate of the Adam optimizer
early_stop_pat	$[0, 20]$ with $\Delta = 5$	epochs without improvement needed for early stopping
n_layers	$[1, 3]$	number of building blocks consisting of convolutional, batch normalization and dropout layer
stride_perc	$[0.5, 1]$ with $\Delta = 0.1$	stride in relation to the kernel size
kernel_size	$[2, 8]$ if $n_{features} \leq 15.000$<br>$[4, 10]$ with $\Delta = 2$ if $15.000 < n_{features} \leq 50.000$<br>$[8, 14]$ with $\Delta = 2$ if $n_{features} > 50.000$	size of the convolutional and max pooling kernels
n_units_fac_lin	$[0.2, 1]$ with $\Delta = 0.05$ if $n_{features} \leq 15.000$<br>$[0.2, 0.5]$ with $\Delta = 2$ if $n_{features} > 15.000$	neurons in the fully-connected layer after the convolutional network part in relation to the hidden output size
	LCNN	
dropout	$[0, 0.5]$ with $\Delta = 0.1$	dropout rate for dropout layers
learning_rate	$[10^{-6}, 10^{-5}, 10^{-4}, 10^{-3}, 10^{-2}, 10^{-1}]$	learning rate of the Adam optimizer
early_stop_pat	$[0, 10]$ with $\Delta = 10$	epochs without improvement needed for early stopping
stride_perc	$[0.5, 1]$ with $\Delta = 0.1$	stride in relation to the kernel size
kernel_size	$[6, 14]$ with $\Delta = 2$ if $n_{features} \leq 15.000$<br>$[3, 7]$ with $\Delta = 1$ if $n_{features} > 20.000$	size of the locally connected kernels, for $n_{features} > 20.000$ as exponent to the basis 2
n_layers	$[1, 3]$	number of building blocks consisting of the fully-connected part after the locally connected layer
n_units_fac_lin	$[0.1, 0.7]$ with $\Delta = 0.05$	neurons in the fully-connected layer after the convolutional network part in relation to the hidden output size
perc_dec	$[0.2, 0.5]$ with $\Delta = 0.05$	percentage decrease of number of neurons per building block

Table S4. Results of Bayes A, Bayes B and Bayes C for synthetic data: For each heritability ($h = 0.7$, $h = 0.85$, $h = 0.95$) and simulation setting (*A* to *L*), the table shows the explained variances (mean and standard deviation on outer folds of nested cross-validation) achieved by the corresponding model.

	Simulation	Bayes A	Bayes B	Bayes C
$h = 0.7$	*A* (#100)	0.099 ± 0.011	0.116 ± 0.008	0.096 ± 0.011
	B (#500)	0.450 ± 0.059	0.446 ± 0.055	0.300 ± 0.076
	C (#1000)	0.495 ± 0.034	0.492 ± 0.036	0.424 ± 0.062
	D (#2000)	0.507 ± 0.013	0.516 ± 0.011	0.483 ± 0.011
	E (MultWeak)	0.354 ± 0.004	0.353 ± 0.006	0.266 ± 0.004
	F (MultStrong)	0.231 ± 0.002	0.236 ± 0.005	0.223 ± 0.009
	G (SkewedWeak)	0.512 ± 0.034	0.509 ± 0.033	0.432 ± 0.034
	H (SkewedStrong)	0.479 ± 0.008	0.483 ± 0.010	0.430 ± 0.007
	I (Add5)	0.369 ± 0.048	0.384 ± 0.049	0.354 ± 0.047
	J (Add20)	0.393 ± 0.021	0.400 ± 0.018	0.380 ± 0.018
	K (Add50)	0.551 ± 0.029	0.549 ± 0.030	0.548 ± 0.029
	L (Add100)	0.477 ± 0.037	0.478 ± 0.035	0.477 ± 0.035
$h = 0.85$	*A* (#100)	0.278 ± 0.120	0.275 ± 0.121	0.273 ± 0.124
	B (#500)	0.489 ± 0.048	0.437 ± 0.115	0.338 ± 0.027
	C (#1000)	0.561 ± 0.061	0.560 ± 0.059	0.489 ± 0.070
	D (#2000)	0.659 ± 0.004	0.674 ± 0.003	0.628 ± 0.007
	E (MultWeak)	0.442 ± 0.032	0.439 ± 0.029	0.382 ± 0.005
	F (MultStrong)	0.177 ± 0.043	0.184 ± 0.047	0.174 ± 0.051
	G (SkewedWeak)	0.561 ± 0.023	0.559 ± 0.029	0.520 ± 0.036
	H (SkewedStrong)	0.611 ± 0.009	0.612 ± 0.009	0.493 ± 0.013
	I (Add5)	0.633 ± 0.012	0.638 ± 0.006	0.576 ± 0.032
	J (Add20)	0.472 ± 0.037	0.483 ± 0.037	0.463 ± 0.038
	K (Add50)	0.590 ± 0.021	0.593 ± 0.022	0.589 ± 0.021
	L (Add100)	0.568 ± 0.035	0.572 ± 0.034	0.564 ± 0.035
$h = 0.95$	*A* (#100)	0.214 ± 0.061	0.216 ± 0.071	0.211 ± 0.065
	B (#500)	0.531 ± 0.047	0.533 ± 0.047	0.465 ± 0.030
	C (#1000)	0.656 ± 0.011	0.659 ± 0.007	0.534 ± 0.033
	D (#2000)	0.736 ± 0.012	0.758 ± 0.013	0.716 ± 0.014
	E (MultWeak)	0.508 ± 0.019	0.513 ± 0.019	0.470 ± 0.020
	F (MultStrong)	0.567 ± 0.049	0.571 ± 0.048	0.557 ± 0.053
	G (SkewedWeak)	0.637 ± 0.006	0.636 ± 0.009	0.561 ± 0.012
	H (SkewedStrong)	0.640 ± 0.026	0.650 ± 0.024	0.549 ± 0.049
	I (Add5)	0.517 ± 0.028	0.523 ± 0.030	0.477 ± 0.015
	J (Add20)	0.680 ± 0.038	0.707 ± 0.038	0.687 ± 0.044
	K (Add50)	0.571 ± 0.036	0.577 ± 0.037	0.566 ± 0.034
	L (Add100)	0.639 ± 0.017	0.640 ± 0.024	0.640 ± 0.018

Table S5. Results of Bayes A, Bayes B and Bayes C for real-world data: For each species-phenotype combination, the table shows the explained variances (mean and standard deviation on outer folds of nested cross-validation) achieved by the corresponding model.

	Phenotype	Bayes A	Bayes B	Bayes C
A. thaliana	DTF1	0.628 ± 0.028	0.629 ± 0.028	0.629 ± 0.030
	RL	0.520 ± 0.007	0.518 ± 0.008	0.516 ± 0.005
	Diameter	-0.030 ± 0.014	-0.009 ± 0.012	-0.000 ± 0.009
	FT10	0.647 ± 0.020	0.649 ± 0.019	0.645 ± 0.021
corn	PWC	0.481 ± 0.015	0.482 ± 0.015	0.481 ± 0.015
	Yield	0.276 ± 0.007	0.277 ± 0.006	0.276 ± 0.009
soy	MatG	0.587 ± 0.050	0.588 ± 0.051	0.586 ± 0.046
	Yield	0.126 ± 0.014	0.118 ± 0.032	0.128 ± 0.017
	Height	0.474 ± 0.065	0.463 ± 0.067	0.470 ± 0.059

Table S6. Analysis of feature importances of LASSO, ElasticNet, RR-BLUP, Bayes B, RF and XGB for synthetic data with $h = 0.7$: For each simulation configuration and model, the table shows the number of SNPs deemed as important feature for at least one of the outer folds in the nested cross-validation, both, without and with filtering out those which are smaller than one percent of the largest feature importance. Furthermore, the number of background SNPs within the important features are stated as well as the ratio between the found background SNPs and the total amount of important features as percentage value in parentheses, i.e. the True Positive Rate (TPR), again without and with filtering. For the causal SNPs, we show the number of causal SNPs deemed important by each algorithm and in brackets the ranking of the causal SNPs within the important features. For configurations *J*, *K* and *L*, we give the number of causal SNPs within the k (total number of causal SNPs) most important features.
Where *A*: #100, *B*: #500, *C*: #1000, *D*: #2000, *E*: MultWeak, *F*: MultStrong, *G*: SkewedWeak, *H*: SkewedStrong, *I*: Add5, *J*: Add20, *K*: Add50, *L*: Add100

	LASSO		ElasticNet		RR-BLUP		Bayes B		RF		XGB	
**Sim**	no filter	1% filter	no filter	1% filter	no filter	1% filter	no filter	1% filter	no filter	1% filter	no filter	1% filter
	**important features**											
A	38	32	935	841	2099	2099	2333	2333	2052	2048	2	2
B	95	43	102	49	2122	2122	2211	4	2460	1057	300	203
C	213	79	221	84	2159	2159	2195	6	1932	593	1403	1401
D	427	160	653	243	2137	2137	2074	109	2357	140	2274	1176
E	347	218	1973	1578	2125	2125	2156	91	2094	2094	523	523
F	144	114	1650	1441	2125	2125	2114	837	2228	2228	2310	2310
G	273	108	274	105	2095	2095	2137	8	2251	743	2460	1591
H	177	80	182	83	2120	2120	2108	17	2198	870	2215	1471
I	345	250	1451	942	2094	2094	2156	411	1938	1938	2139	2093
J	159	131	977	755	2076	2076	2139	1151	2080	2080	1764	1100
K	307	284	2107	2099	2093	2093	2156	2156	2374	1363	2074	634
L	288	268	2003	1810	2066	2066	2101	2101	2202	2202	1959	442
	**background SNPs**											
A	10 (26%)	10 (31%)	112 (12%)	103 (12%)	229 (11%)	229 (11%)	262 (11%)	262 (11%)	211 (10%)	210 (10%)	1 (50%)	1 (50%)
B	14 (15%)	11 (26%)	17 (17%)	11 (22%)	239 (11%)	239 (11%)	252 (11%)	1 (25%)	294 (12%)	129 (12%)	48 (16%)	36 (18%)
C	47 (22%)	21 (27%)	47 (21%)	24 (29%)	234 (11%)	234 (11%)	269 (12%)	3 (50%)	223 (12%)	73 (12%)	185 (13%)	185 (13%)
D	87 (20%)	60 (38%)	116 (18%)	76 (31%)	244 (11%)	244 (11%)	273 (13%)	50 (46%)	276 (12%)	21 (15%)	306 (13%)	183 (16%)
E	53 (15%)	37 (17%)	218 (11%)	183 (12%)	248 (12%)	248 (12%)	226 (10%)	18 (20%)	249 (12%)	249 (12%)	91 (17%)	91 (17%)
F	27 (19%)	24 (21%)	204 (12%)	182 (13%)	231 (11%)	231 (11%)	243 (11%)	103 (12%)	247 (11%)	247 (11%)	269 (12%)	269 (12%)
G	46 (17%)	20 (19%)	45 (16%)	18 (17%)	231 (11%)	231 (11%)	250 (12%)	1 (12%)	244 (11%)	75 (10%)	282 (11%)	181 (11%)
H	29 (16%)	16 (20%)	30 (16%)	16 (19%)	234 (11%)	234 (11%)	233 (11%)	5 (29%)	242 (11%)	98 (11%)	251 (11%)	174 (12%)
I	45 (13%)	33 (13%)	164 (11%)	108 (11%)	239 (11%)	239 (11%)	239 (11%)	60 (15%)	217 (11%)	217 (11%)	259 (12%)	256 (12%)
J	40 (25%)	34 (26%)	123 (13%)	102 (14%)	232 (11%)	232 (11%)	237 (11%)	143 (12%)	248 (12%)	248 (12%)	216 (12%)	145 (13%)
K	44 (14%)	40 (14%)	220 (10%)	220 (10%)	246 (12%)	246 (12%)	219 (10%)	219 (10%)	252 (11%)	144 (11%)	245 (12%)	74 (12%)
L	46 (16%)	43 (16%)	208 (10%)	186 (10%)	226 (11%)	226 (11%)	217 (10%)	217 (10%)	220 (10%)	220 (10%)	189 (10%)	55 (12%)
	**causal SNPs**											
A	1/1 [1]		1/1 [1]		0/1 [-]		1/1 [2]		1/1 [1]		1/1 [1]	
B	1/1 [1]		1/1 [1]		0/1 [-]		1/1 [1]		1/1 [1]		1/1 [1]	
C	1/1 [1]		1/1 [1]		0/1 [-]		1/1 [1]		1/1 [1]		1/1 [2]	
D	1/1 [1]		1/1 [1]		0/1 [-]		1/1 [1]		1/1 [1]		1/1 [1]	
E	2/2 [1,2]		2/2 [1,2]		0/2 [-]		2/2 [1, 2]		2/2 [1, 2]		2/2 [4, 6]	
F	2/2 [1,3]		2/2 [1,2]		0/2 [-]		2/2 [1, 2]		2/2 [2, 24]		2/2 [1, 51]	
G	1/1 [1]		1/1 [1]		0/1 [-]		1/1 [1]		1/1 [1]		1/1 [4]	
H	1/1 [1]		1/1 [1]		0/1 [-]		1/1 [1]		1/1 [2]		1/1 [1]	
I	4/5 [1,2,26,28]		5/5 [1,2,13,36,926]		0/5 [-]		5/5 [1, 2, 3, 49, 696]		5/5 [1, 2, 4, 6, 766]		5/5 [52, 74, 77, 142, 651]	
J	16/20 [12 in top20]		19/20 [12 in top20]		0/20 [0 in top20]		20/20 [14 in top20]		12/20 [0 in top20]		16/20 [0 in top20]	
K	17/50 [6 in top50]		43/50 [10 in top50]		0/50 [0 in top50]		41/50 [10 in top50]		26/50 [1 in top50]		28/50 [2 in top50]	
L	20/100 [8 in top100]		63/100 [14 in top100]		7/100 [0 in top100]		65/100 [15 in top100]		46/100 [3 in top100]		27/100 [6 in top100]	

Table S7. Analysis of feature importances of LASSO, ElasticNet, RR-BLUP, Bayes B, RF and XGB for synthetic data with $h = 0.85$: For each simulation configuration and model, the table shows the number of SNPs deemed as important feature for at least one of the outer folds in the nested cross-validation, both, without and with filtering out those which are smaller than one percent of the largest feature importance. Furthermore, the number of background SNPs within the important features are stated as well as the ratio between the found background SNPs and the total amount of important features as percentage value in parentheses, i.e. the True Positive Rate (TPR), again without and with filtering. For the causal SNPs, we show the number of causal SNPs deemed important by each algorithm and in brackets the ranking of the causal SNPs within the important features. For configurations *J*, *K* and *L*, we give the number of causal SNPs within the k (total number of causal SNPs) most important features.
Where *A*: #100, *B*: #500, *C*: #1000, *D*: #2000, *E*: MultWeak, *F*: MultStrong, *G*: SkewedWeak, *H*: SkewedStrong, *I*: Add5, *J*: Add20, *K*: Add50, *L*: Add100

	LASSO		ElasticNet		RR-BLUP		Bayes B		RF		XGB	
**Sim**	no filter	1% filter	no filter	1% filter	no filter	1% filter	no filter	1% filter	no filter	1% filter	no filter	1% filter
	**important features**											
A	43	31	359	157	2061	2061	2336	2336	2160	1987	45	45
B	149	77	598	198	2087	2087	2199	13	2158	992	202	202
C	413	169	421	170	2101	2101	2079	35	2150	323	956	153
D	556	156	593	171	2043	2043	2120	91	1790	335	2317	1055
E	284	198	740	379	2113	2113	2117	181	2098	2098	1886	473
F	133	125	1351	1213	2164	2164	2100	2100	2095	2095	1964	1533
G	305	147	1187	371	2060	2060	2112	38	2246	837	1826	1272
H	253	88	256	90	2094	2094	2115	17	2099	768	863	572
I	315	190	299	179	2108	2108	2193	94	2020	2008	1419	353
J	462	417	1723	1551	2076	2076	2054	2054	1946	1946	2435	2042
K	490	452	1991	1916	2075	2075	2045	2045	1676	1676	1945	457
L	382	355	2056	2056	2056	2056	2094	2094	1775	1775	2626	1368
	**background SNPs**											
A	3 (7%)	2 (6%)	40 (11%)	17 (11%)	233 (11%)	233 (11%)	263 (11%)	263 (11%)	239 (11%)	219 (11%)	4 (9%)	4 (9%)
B	23 (15%)	17 (22%)	80 (13%)	34 (17%)	212 (10%)	212 (10%)	229 (10%)	5 (38%)	231 (11%)	104 (10%)	34 (17%)	34 (17%)
C	95 (23%)	57 (34%)	97 (23%)	58 (34%)	228 (11%)	228 (11%)	273 (13%)	22 (63%)	256 (12%)	59 (18%)	134 (14%)	23 (15%)
D	105 (19%)	51 (33%)	110 (19%)	56 (33%)	252 (12%)	252 (12%)	288 (14%)	48 (53%)	237 (13%)	55 (16%)	326 (14%)	185 (18%)
E	59 (21%)	47 (24%)	119 (16%)	79 (21%)	242 (11%)	242 (11%)	244 (12%)	47 (26%)	228 (11%)	228 (11%)	219 (12%)	61 (13%)
F	22 (17%)	21 (17%)	158 (12%)	145 (12%)	261 (12%)	261 (12%)	227 (11%)	227 (11%)	247 (12%)	247 (12%)	220 (11%)	179 (12%)
G	60 (20%)	42 (29%)	155 (13%)	58 (16%)	256 (12%)	256 (12%)	256 (12%)	19 (50%)	262 (12%)	99 (12%)	236 (13%)	179 (14%)
H	46 (18%)	25 (28%)	46 (18%)	24 (27%)	221 (11%)	221 (11%)	259 (12%)	9 (53%)	255 (12%)	106 (14%)	136 (16%)	84 (15%)
I	52 (17%)	35 (18%)	52 (17%)	37 (21%)	248 (12%)	248 (12%)	262 (12%)	29 (31%)	207 (10%)	207 (10%)	174 (12%)	54 (15%)
J	74 (16%)	70 (17%)	194 (11%)	183 (12%)	246 (12%)	246 (12%)	245 (12%)	245 (12%)	243 (12%)	243 (12%)	250 (10%)	208 (10%)
K	72 (15%)	68 (15%)	221 (11%)	219 (11%)	242 (12%)	242 (12%)	220 (11%)	220 (11%)	173 (10%)	173 (10%)	235 (12%)	69 (15%)
L	59 (15%)	56 (16%)	234 (11%)	234 (11%)	259 (13%)	259 (13%)	237 (11%)	237 (11%)	206 (12%)	206 (12%)	296 (11%)	157 (11%)
	**causal SNPs**											
A	1/1 [1]		1/1 [1]		0/1 [-]		1/1 [1]		1/1 [1]		1/1 [1]	
B	1/1 [1]		1/1 [1]		0/1 [-]		1/1 [1]		1/1 [1]		1/1 [2]	
C	1/1 [1]		1/1 [1]		0/1 [-]		1/1 [1]		1/1 [1]		1/1 [1]	
D	1/1 [1]		1/1 [1]		0/1 [-]		1/1 [1]		1/1 [1]		1/1 [1]	
E	2/2 [1,2]		2/2 [1,2]		0/2 [-]		2/2 [1,2]		2/2 [1,3]		2/2 [5,8]	
F	2/2 [2,8]		2/2 [4,44]		0/2 [-]		2/2 [1,4]		2/2 [47,48]		2/2 [22,82]	
G	1/1 [1]		1/1 [1]		0/1 [-]		1/1 [1]		1/1 [1]		1/1 [1]	
H	1/1 [1]		1/1 [1]		0/1 [-]		1/1 [1]		1/1 [1]		1/1 [2]	
I	5/5 [1,2,3,4,5]		5/5 [1,2,3,4,5]		0/5 [-]		5/5 [1,2,3,4,5]		5/5 [2,18,20,67,145]		5/5 [13,23,29,32,86]	
J	17/20 [12 in top20]		19/20 [7 in top20]		0/20 [0 in top20]		19/20 [11 in top20]		18/20 [2 in top20]		15/20 [0 in top20]	
K	24/50 [10 in top50]		38/50 [11 in top50]		0/50 [0 in top50]		41/50 [11 in top50]		20/50 [1 in top50]		25/50 [1 in top50]	
L	24/100 [10 in top100]		61/100 [14 in top100]		0/100 [0 in top100]		59/100 [15 in top100]		35/100 [3 in top100]		28/100 [2 in top100]	

Table S8. Analysis of feature importances of LASSO, ElasticNet, RR-BLUP, Bayes B, RF and XGB for synthetic data with $h = 0.95$: For each simulation configuration and model, the table shows the number of SNPs deemed as important feature for at least one of the outer folds in the nested cross-validation, both, without and with filtering those which are smaller than one percent of the largest feature importance. Furthermore, the number of background SNPs within the important features are stated as well as the ratio between the found background SNPs and the total amount of important features as percentage value in parentheses, again without and with filtering. For the causal SNPs, we show the number of causal SNPs deemed important by each algorithm and in brackets the ranking of the causal SNPs within the important features. For configurations *J*, *K* and *L*, we give the number of causal SNPs within the k (total number of causal SNPs) most important features.
Where *A*: #100, *B*: #500, *C*: #1000, *D*: #2000, *E*: MultWeak, *F*: MultStrong, *G*: SkewedWeak, *H*: SkewedStrong, *I*: Add5, *J*: Add20, *K*: Add50, *L*: Add100

	LASSO		ElasticNet		RR-BLUP		Bayes B		RF		XGB	
**Sim**	no filter	1% filter	no filter	1% filter	no filter	1% filter	no filter	1% filter	no filter	1% filter	no filter	1% filter
						**important features**						
A	42	40	733	582	2075	2075	2327	2327	2354	2213	87	87
B	299	187	1466	674	2090	2090	2124	21	1924	1121	890	66
C	531	142	541	148	2085	2085	2134	26	2428	772	1749	459
D	888	293	1004	374	2039	2039	2112	167	2001	510	2479	60
E	333	225	1639	1137	2091	2091	2080	286	1921	1921	1928	792
F	299	238	1595	1131	2082	2082	2094	562	1654	1654	1897	1047
G	404	157	441	186	2076	2076	2072	33	2169	839	2528	1202
H	384	135	387	135	2068	2068	2028	25	2006	548	1993	1118
I	549	425	1775	1533	2060	2060	2105	838	1993	1993	2590	2590
J	619	490	1181	839	2079	2079	2217	569	1812	1592	1593	254
K	748	661	2122	2122	2023	2023	2081	2081	2089	2089	1991	1492
L	516	464	2000	2000	2096	2096	2091	2091	1745	1745	1824	1017
						**background SNPs**						
A	3 (7%)	3 (7%)	90 (12%)	75 (13%)	228 (11%)	228 (11%)	265 (11%)	265 (11%)	258 (11%)	241 (11%)	11 (13%)	11 (13%)
B	43 (14%)	31 (17%)	152 (10%)	84 (12%)	225 (11%)	225 (11%)	243 (11%)	9 (43%)	222 (12%)	141 (13%)	105 (12%)	10 (15%)
C	94 (18%)	41 (29%)	94 (17%)	42 (28%)	237 (11%)	237 (11%)	274 (13%)	15 (58%)	272 (11%)	120 (16%)	232 (13%)	75 (16%)
D	179 (20%)	100 (34%)	186 (19%)	125 (33%)	249 (12%)	249 (12%)	322 (15%)	89 (53%)	243 (12%)	75 (15%)	357 (14%)	15 (25%)
E	58 (17%)	48 (21%)	220 (13%)	174 (15%)	241 (12%)	241 (12%)	260 (12%)	66 (23%)	223 (12%)	223 (12%)	236 (12%)	114 (14%)
F	60 (20%)	51 (21%)	192 (12%)	151 (13%)	254 (12%)	254 (12%)	260 (12%)	121 (22%)	216 (13%)	216 (13%)	221 (12%)	127 (12%)
G	76 (19%)	44 (28%)	82 (19%)	49 (26%)	237 (11%)	237 (11%)	258 (12%)	19 (58%)	240 (11%)	107 (13%)	316 (12%)	170 (14%)
H	65 (17%)	33 (24%)	64 (17%)	33 (24%)	256 (12%)	256 (12%)	217 (11%)	13 (52%)	240 (12%)	74 (14%)	259 (13%)	156 (14%)
I	101 (18%)	86 (20%)	237 (13%)	212 (14%)	255 (12%)	255 (12%)	268 (13%)	155 (18%)	267 (13%)	267 (13%)	333 (13%)	333 (13%)
J	95 (15%)	78 (16%)	147 (12%)	119 (12%)	245 (14%)	245 (12%)	262 (12%)	94 (17%)	208 (11%)	185 (12%)	213 (13%)	40 (16%)
K	102 (14%)	94 (14%)	263 (12%)	263 (12%)	251 (12%)	251 (12%)	253 (12%)	253 (12%)	238 (11%)	238 (11%)	244 (12%)	198 (13%)
L	74 (14%)	66 (14%)	244 (12%)	244 (12%)	252 (12%)	252 (12%)	260 (12%)	260 (12%)	209 (12%)	209 (12%)	231 (13%)	145 (14%)
						**causal SNPs**						
A	1/1 [2]		1/1 [2]		0/1 [-]		1/1 [1]		1/1 [1]		1/1 [49]	
B	1/1 [1]		1/1 [1]		0/1 [-]		1/1 [1]		1/1 [1]		1/1 [1]	
C	1/1 [1]		1/1 [1]		0/1 [-]		1/1 [1]		1/1 [1]		1/1 [3]	
D	1/1 [1]		1/1 [1]		0/1 [-]		1/1 [1]		1/1 [3]		1/1 [4]	
E	2/2 [1,2]		2/2 [1,2]		0/2 [-]		2/2 [1,2]		2/2 [1,3]		2/2 [9,18]	
F	2/2 [1,26]		2/2 [1,134]		0/2 [-]		2/2 [1,57]		2/2 [65,273]		2/2 [30,250]	
G	1/1 [1]		1/1 [1]		0/1 [-]		1/1 [1]		1/1 [1]		1/1 [3]	
H	1/1 [1]		1/1 [1]		0/1 [-]		1/1 [1]		1/1 [1]		1/1 [2]	
I	5/5 [2,4,5,9,10]		5/5 [1,3,6,10,17]		0/5 [-]		5/5 [2,4,6,7,10]		5/5 [1,5,21,22,161]		5/5 [5,66,328,693,2195]	
J	19/20 [13 in top20]		20/20 [12 in top20]		0/20 [0 in top20]		20/20 [13 in top20]		17/20 [1 in top20]		19/20 [2 in top20]	
K	33/50 [15 in top50]		46/50 [15 in top50]		0/50 [0 in top50]		45/50 [15 in top50]		34/50 [1 in top50]		28/50 [0 in top50]	
L	44/100 [17 in top100]		70/100 [26 in top100]		2/100 [0 in top100]		67/100 [27 in top100]		31/100 [0 in top100]		43/100 [1 in top100]	

2 SUPPLEMENTARY FIGURES

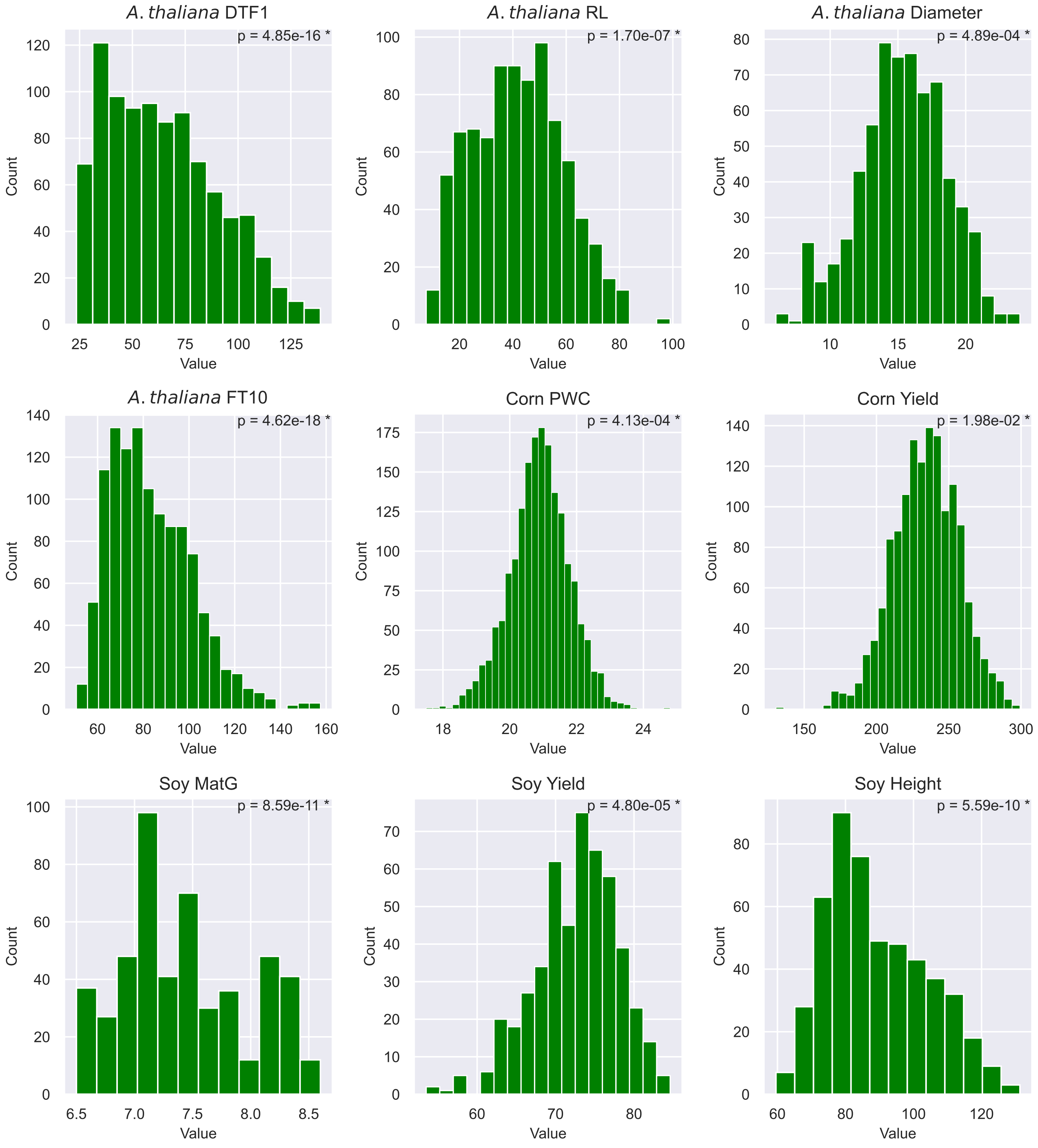

Figure S1. Histograms showing the distributions of all real-world phenotypes: Each subplot shows the distribution of a real-world phenotype. Additionally, for each trait the p-value of the Shapiro-Wilk test is given. Significant p-values (i.e. $p < 0.05$) are marked with an asterisk.

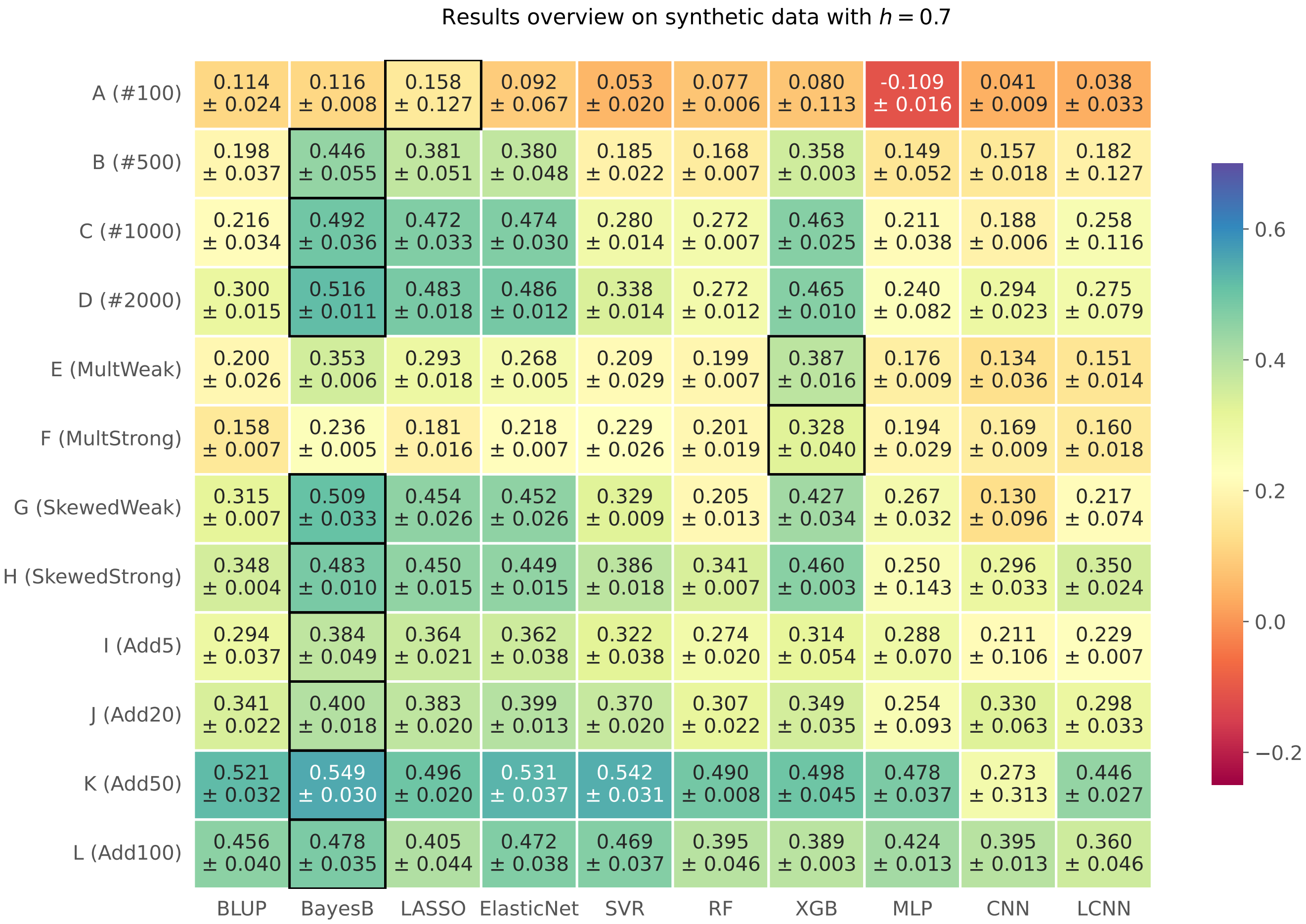

Figure S2. Results on synthetic data with $h = 0.7$ shown in a heatmap: Each cell gives the explained variance ν that the prediction model given on the horizontal axis achieved for the simulation configuration specified on the vertical axis. The color of each cell ranging from dark red to dark blue represents the prediction performance. The best result for each simulated phenotype is highlighted by a black frame around the cell.

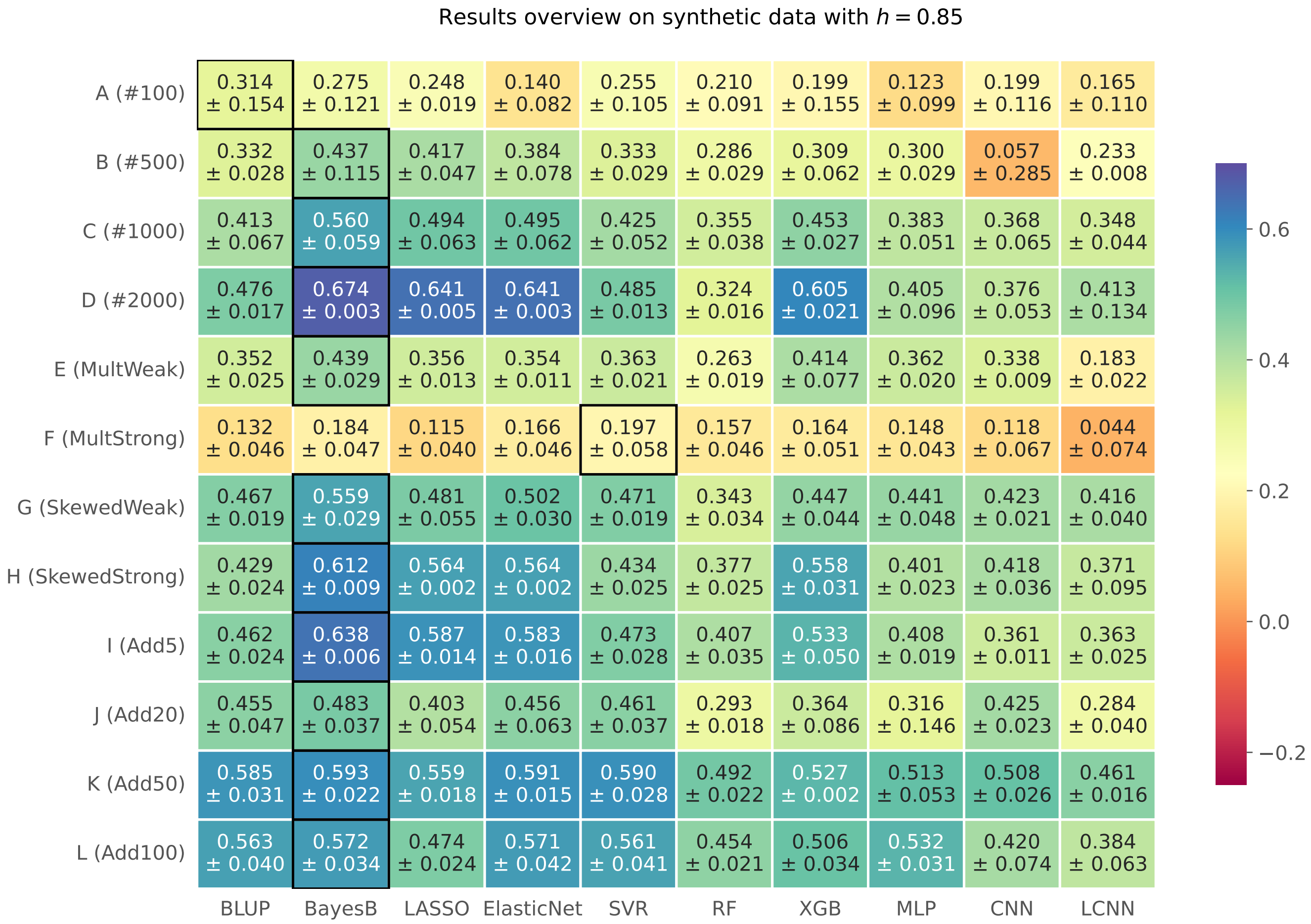

Figure S3. Results on synthetic data with $h = 0.85$ shown in a heatmap: Each cell gives the explained variance ν that the prediction model given on the horizontal axis achieved for the simulation configuration specified on the vertical axis. The color of each cell ranging from dark red to dark blue represents the prediction performance. The best result for each simulated phenotype is highlighted by a black frame around the cell.

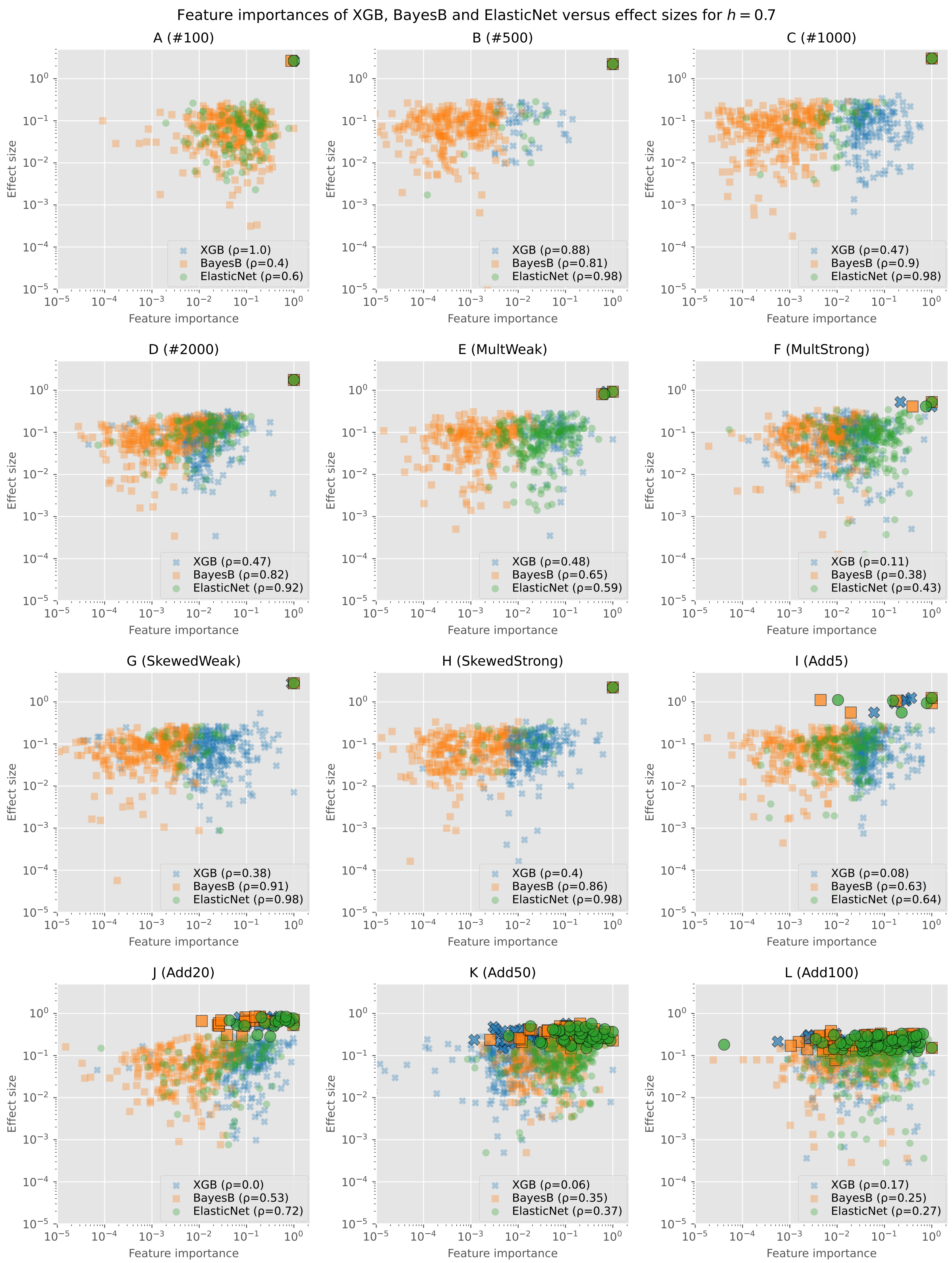

Figure S4. Min-max normalized feature importances of BayesB, ElasticNet and XGB in comparison with effect sizes on synthetic data for $h = 0.7$: Each subplot shows the results of one of the simulation configurations on a logarithmic scale. Only SNPs for which both the effect size as well as the feature importance are not zero are shown. Causal SNPs are highlighted by a larger marker size and a black frame. The legend additionally gives the Pearson correlation coefficient of the effect sizes and feature importances.

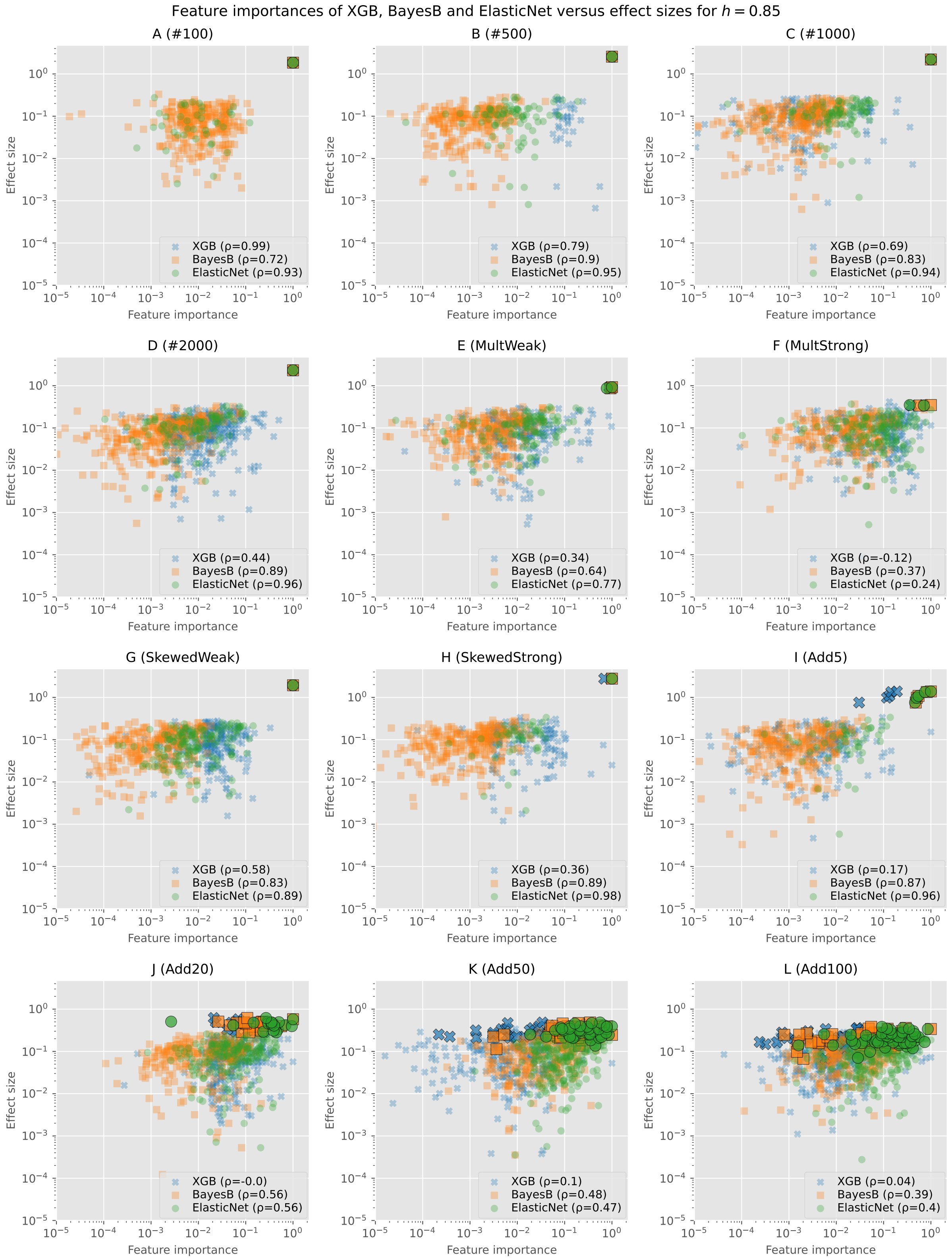

Figure S5. Min-max normalized feature importances of BayesB, ElasticNet and XGB in comparison with effect sizes on synthetic data for $h = 0.85$: Each subplot shows the results of one of the simulation configurations on a logarithmic scale. Only SNPs for which both the effect size as well as the feature importance are not zero are shown. Causal SNPs are highlighted by a larger marker size and a black frame. The legend additionally gives the Pearson correlation coefficient of the effect sizes and feature importances.

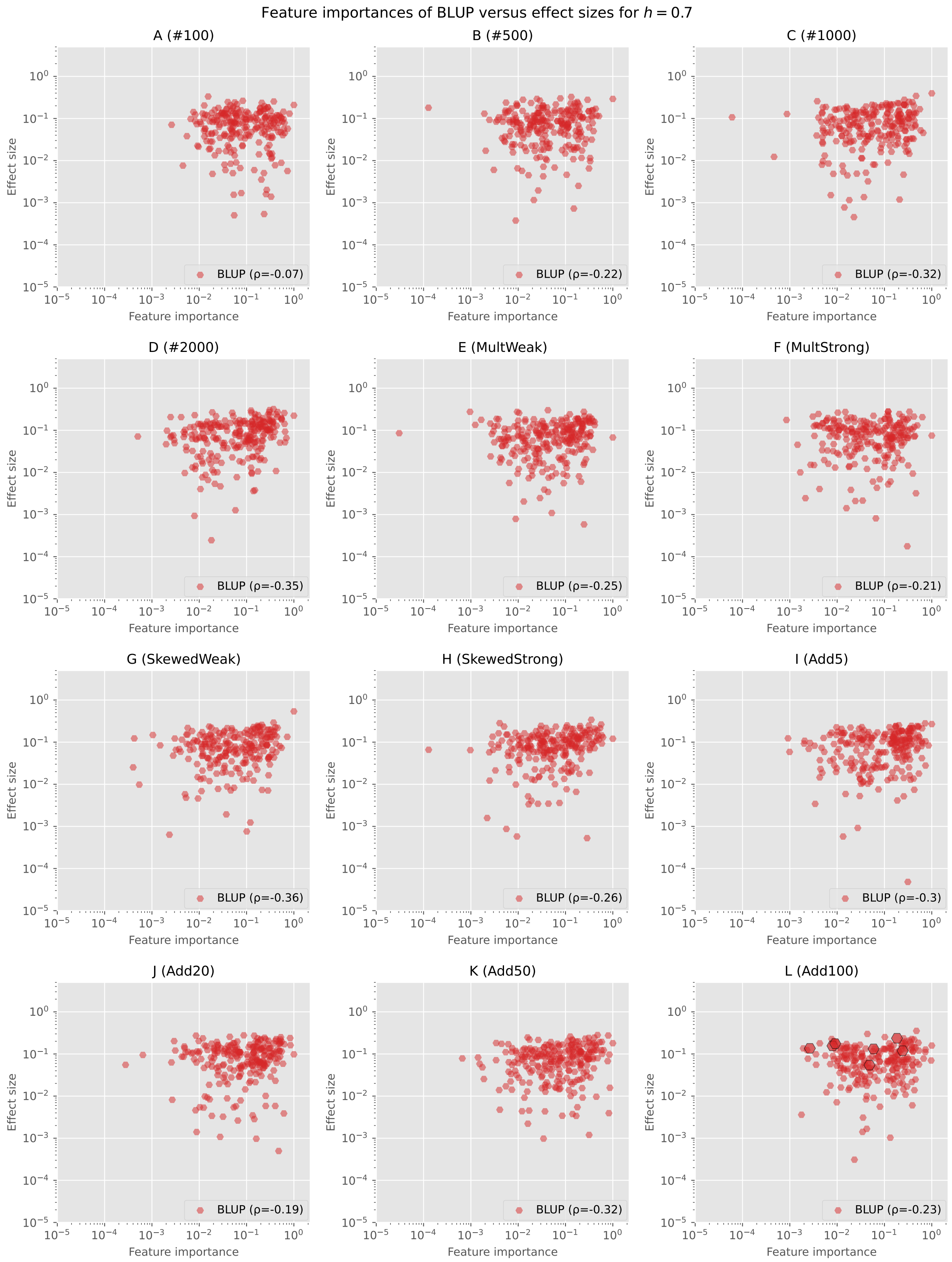

Figure S6. Min-max normalized feature importances of RR-BLUP with effect sizes on synthetic data for $h = 0.7$: Each subplot shows the results of one of the simulation configurations on a logarithmic scale. Only SNPs for which both the effect size as well as the feature importance are not zero are shown. Causal SNPs are highlighted by a larger marker size and a black frame. The legend additionally gives the Pearson correlation coefficient of the effect sizes and feature importances.

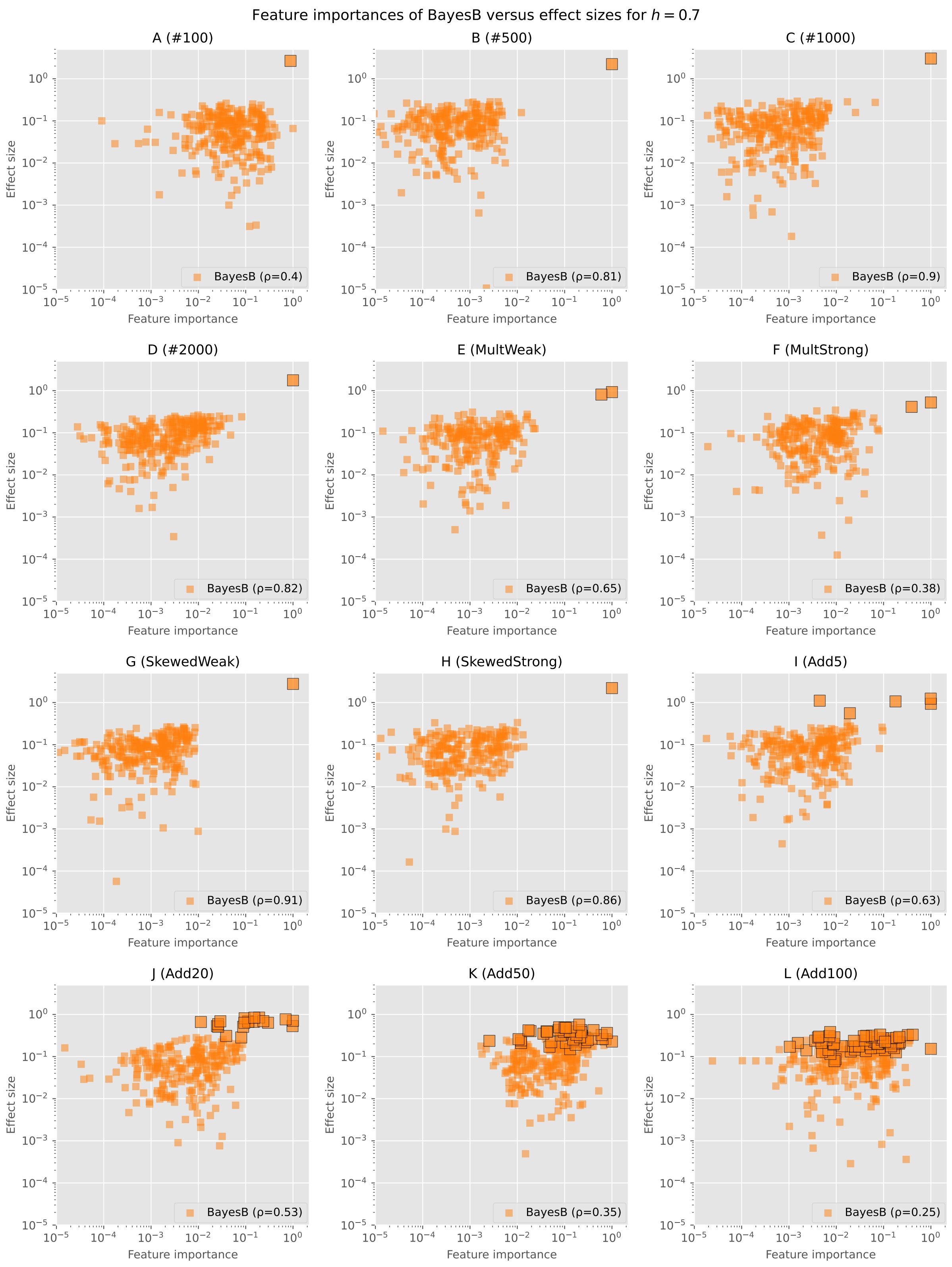

Figure S7. Min-max normalized feature importances of BayesB with effect sizes on synthetic data for $h = 0.7$: Each subplot shows the results of one of the simulation configurations on a logarithmic scale. Only SNPs for which both the effect size as well as the feature importance are not zero are shown. Causal SNPs are highlighted by a larger marker size and a black frame. The legend additionally gives the Pearson correlation coefficient of the effect sizes and feature importances.

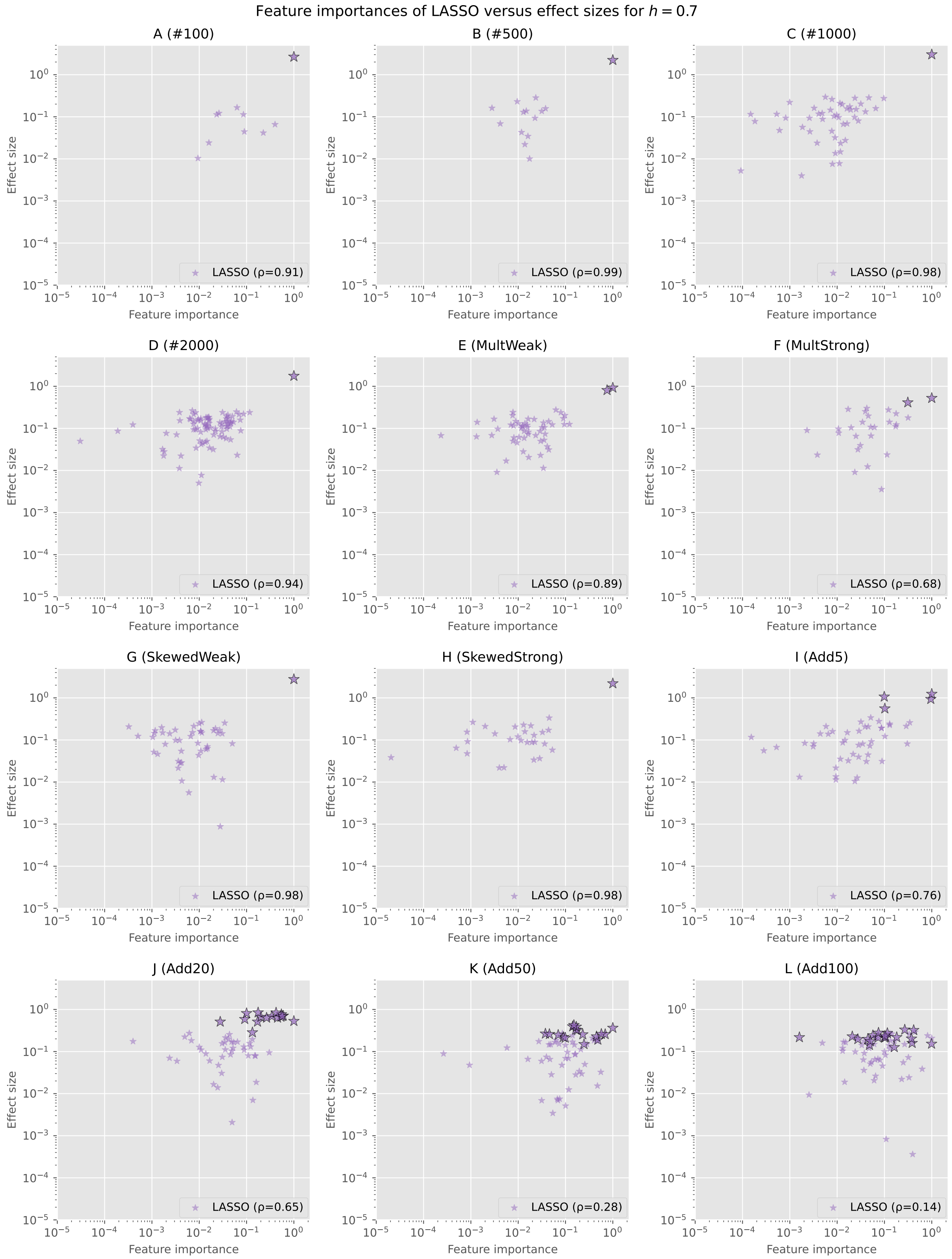

Figure S8. Min-max normalized feature importances of LASSO with effect sizes on synthetic data for $h = 0.7$: Each subplot shows the results of one of the simulation configurations on a logarithmic scale. Only SNPs for which both the effect size as well as the feature importance are not zero are shown. Causal SNPs are highlighted by a larger marker size and a black frame. The legend additionally gives the Pearson correlation coefficient of the effect sizes and feature importances.

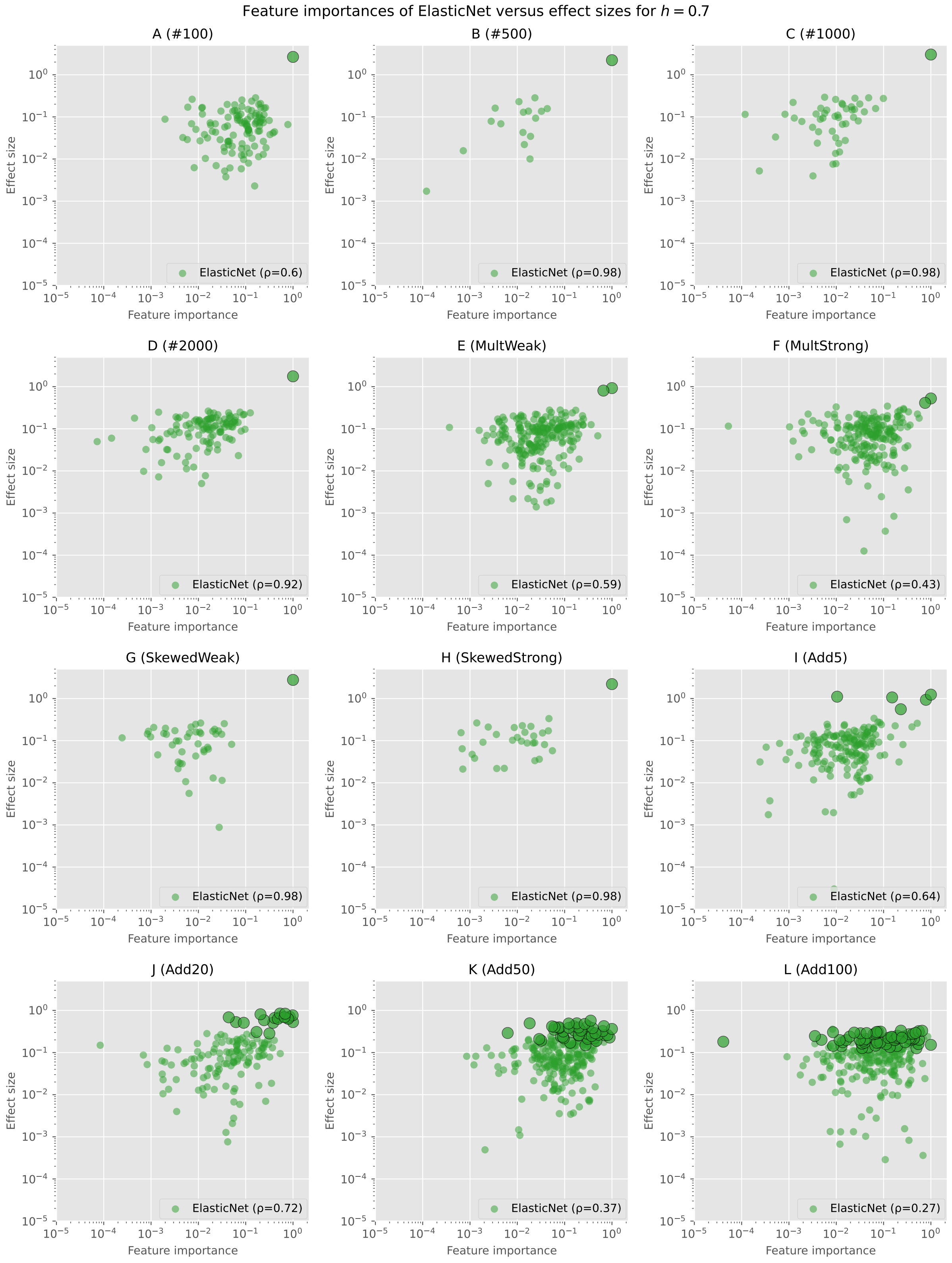

Figure S9. Min-max normalized feature importances of ElasticNet with effect sizes on synthetic data for $h = 0.7$: Each subplot shows the results of one of the simulation configurations on a logarithmic scale. Only SNPs for which both the effect size as well as the feature importance are not zero are shown. Causal SNPs are highlighted by a larger marker size and a black frame. The legend additionally gives the Pearson correlation coefficient of the effect sizes and feature importances.

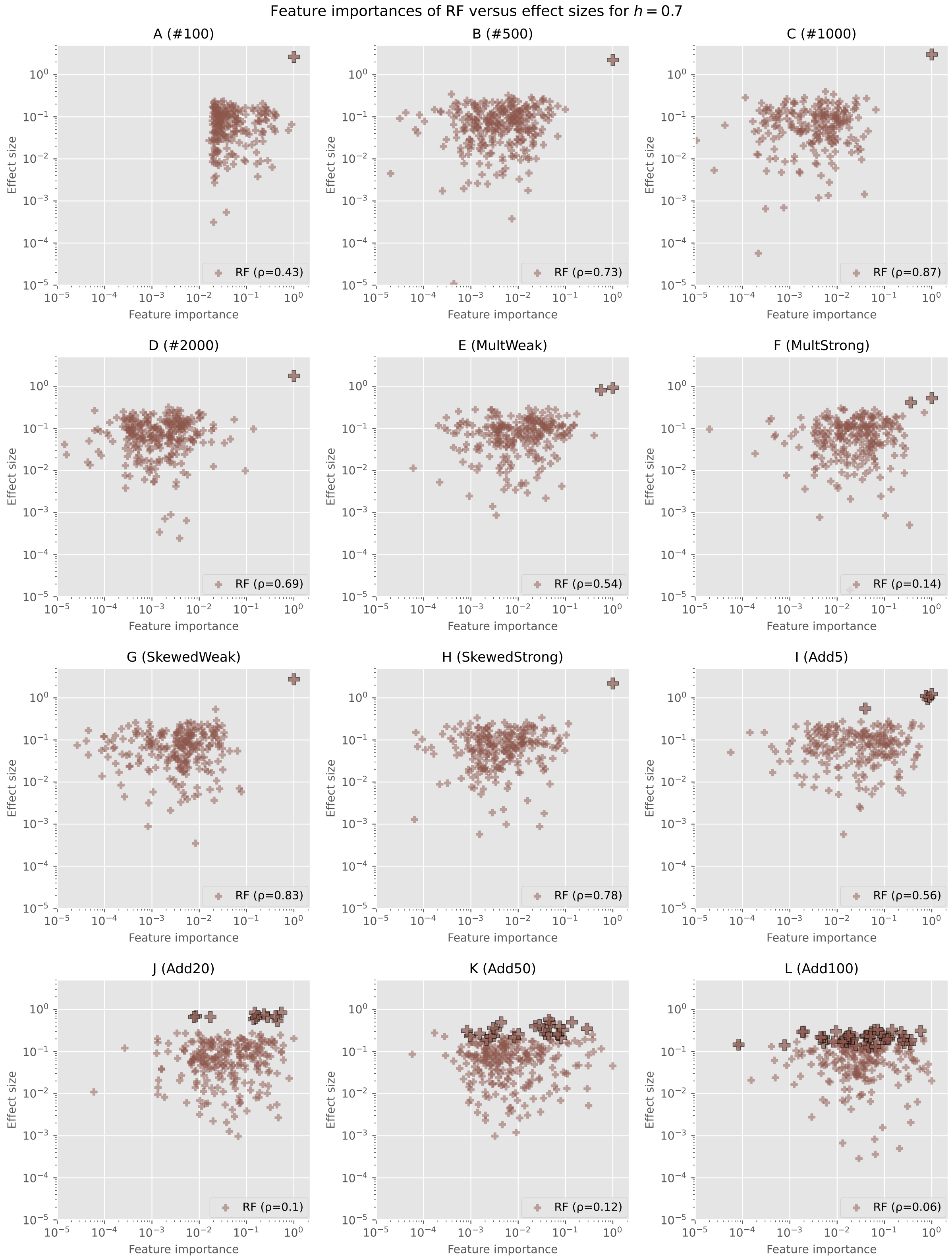

Figure S10. Min-max normalized feature importances of RF with effect sizes on synthetic data for $h = 0.7$: Each subplot shows the results of one of the simulation configurations on a logarithmic scale. Only SNPs for which both the effect size as well as the feature importance are not zero are shown. Causal SNPs are highlighted by a larger marker size and a black frame. The legend additionally gives the Pearson correlation coefficient of the effect sizes and feature importances.

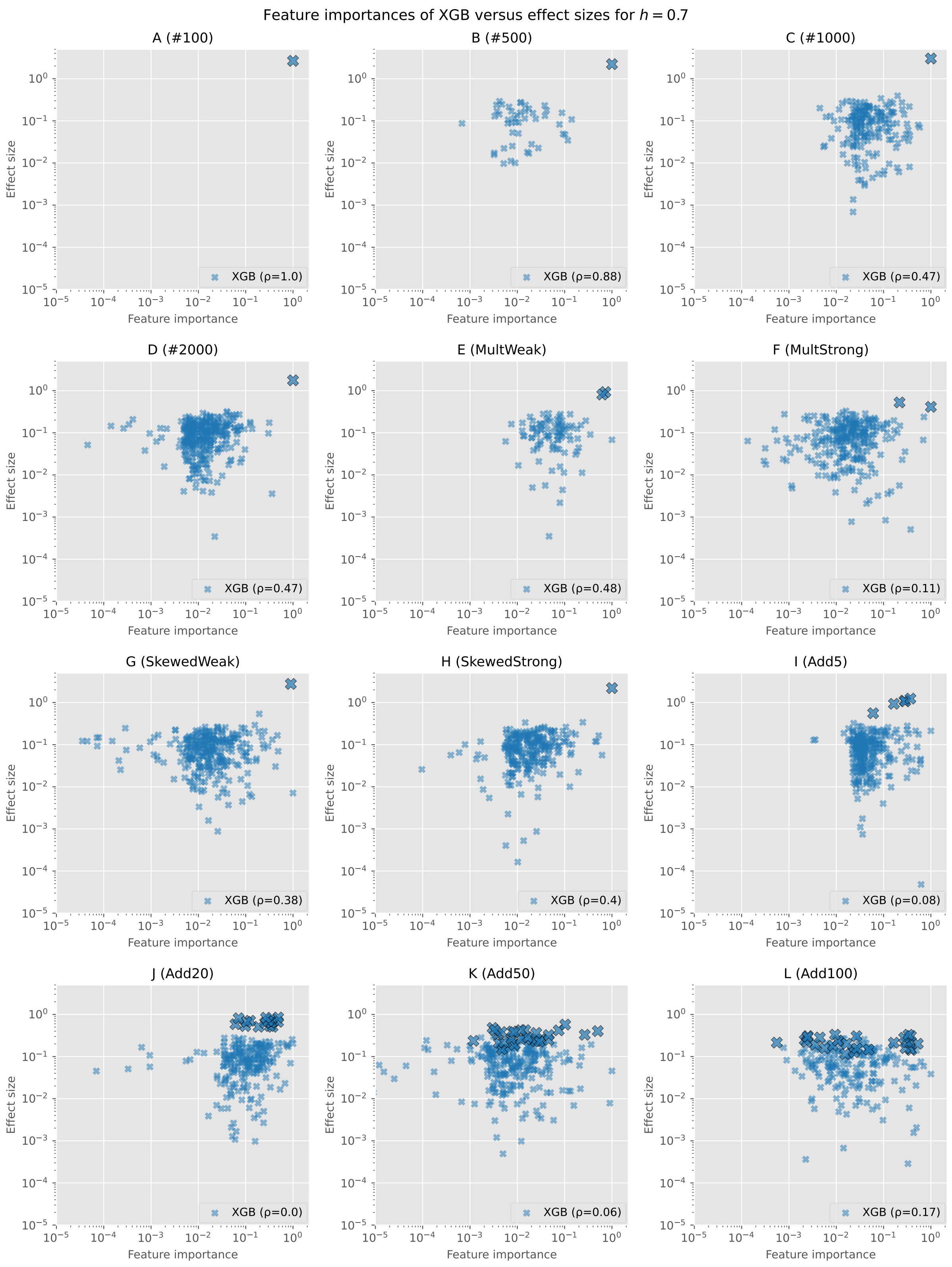

Figure S11. Min-max normalized feature importances of XGB with effect sizes on synthetic data for $h = 0.7$: Each subplot shows the results of one of the simulation configurations on a logarithmic scale. Only SNPs for which both the effect size as well as the feature importance are not zero are shown. Causal SNPs are highlighted by a larger marker size and a black frame. The legend additionally gives the Pearson correlation coefficient of the effect sizes and feature importances.

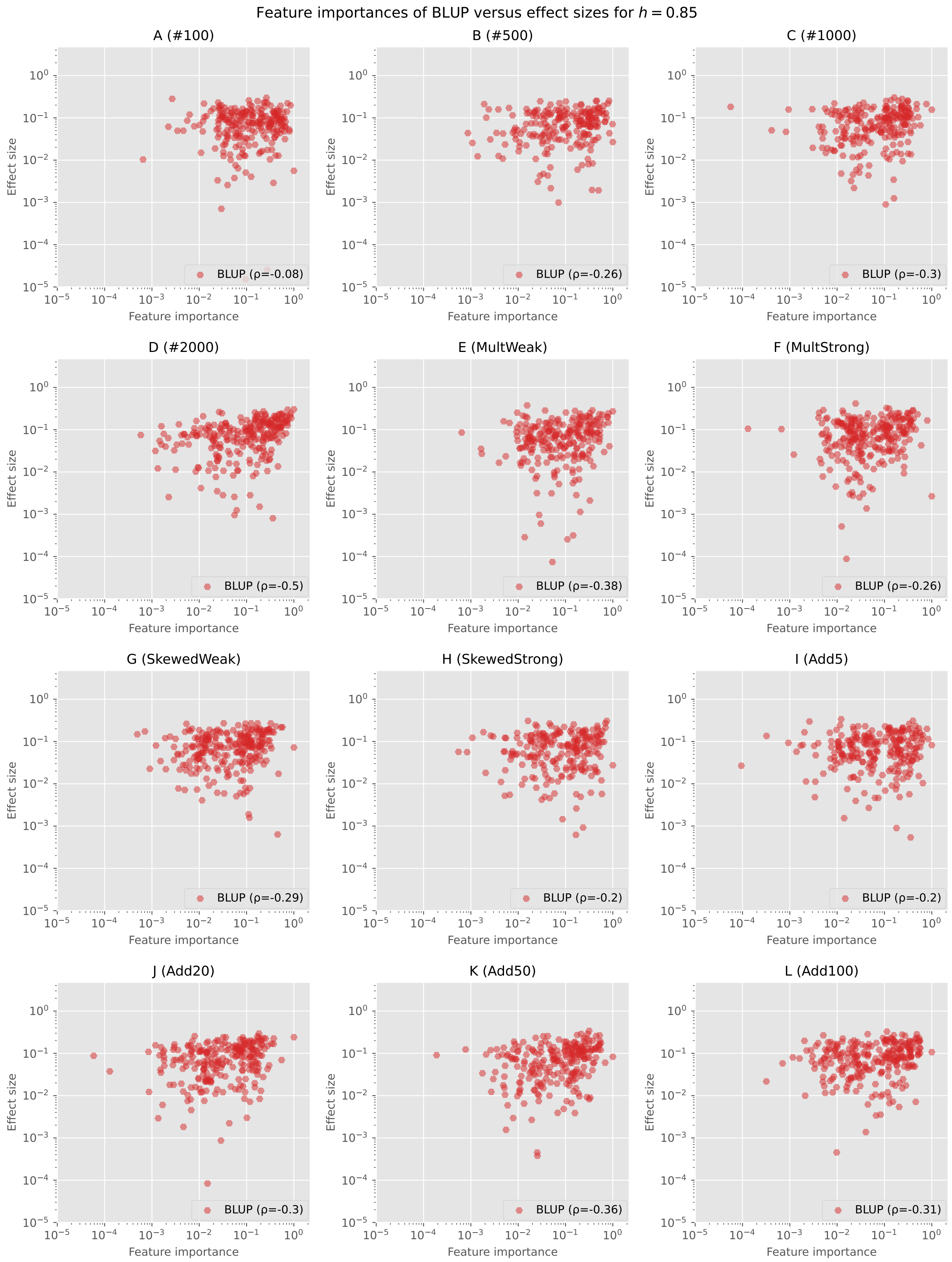

Figure S12. Min-max normalized feature importances of RR-BLUP with effect sizes on synthetic data for $h = 0.85$: Each subplot shows the results of one of the simulation configurations on a logarithmic scale. Only SNPs for which both the effect size as well as the feature importance are not zero are shown. Causal SNPs are highlighted by a larger marker size and a black frame. The legend additionally gives the Pearson correlation coefficient of the effect sizes and feature importances.

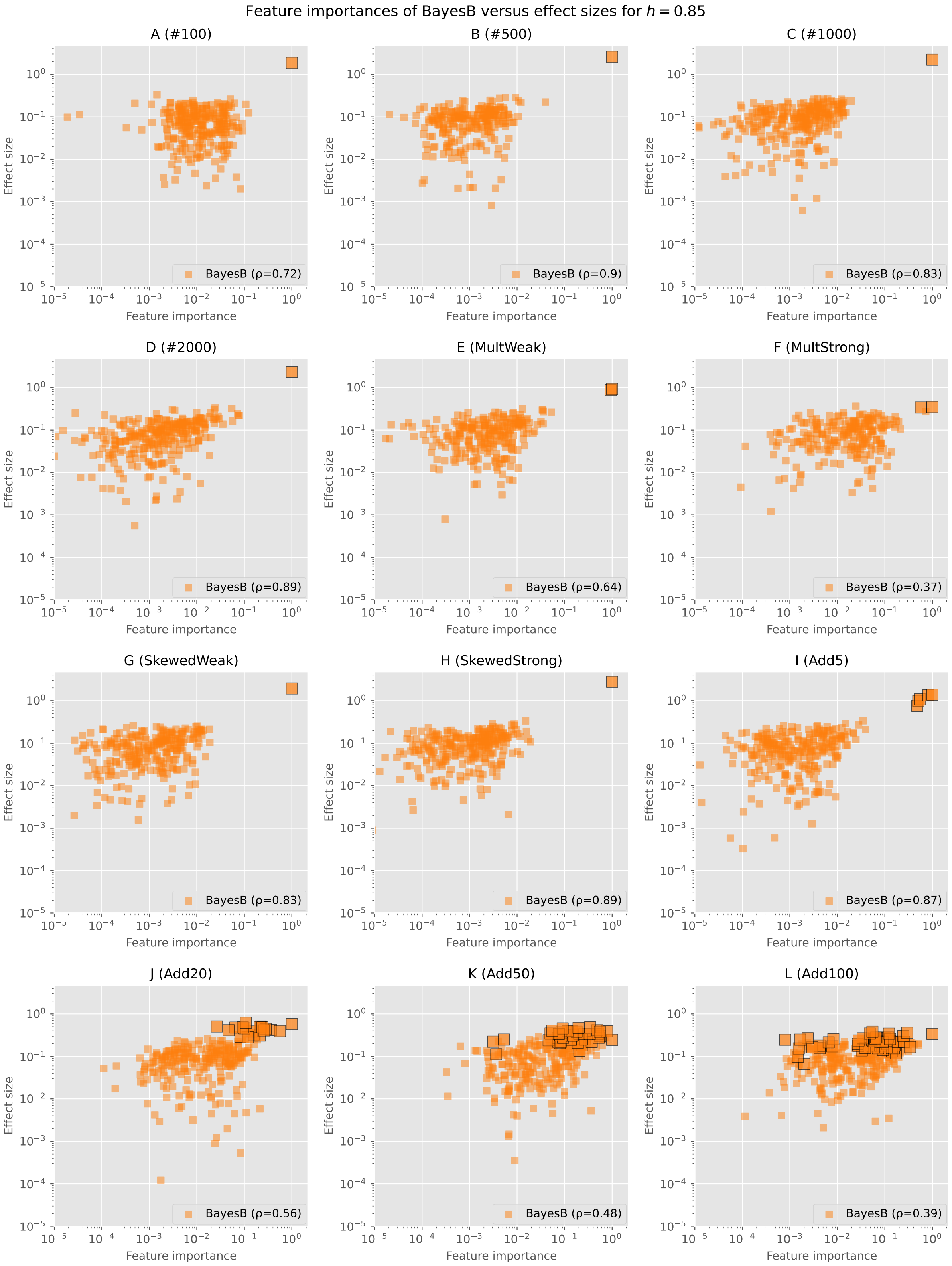

Figure S13. Min-max normalized feature importances of BayesB with effect sizes on synthetic data for $h = 0.85$: Each subplot shows the results of one of the simulation configurations on a logarithmic scale. Only SNPs for which both the effect size as well as the feature importance are not zero are shown. Causal SNPs are highlighted by a larger marker size and a black frame. The legend additionally gives the Pearson correlation coefficient of the effect sizes and feature importances.

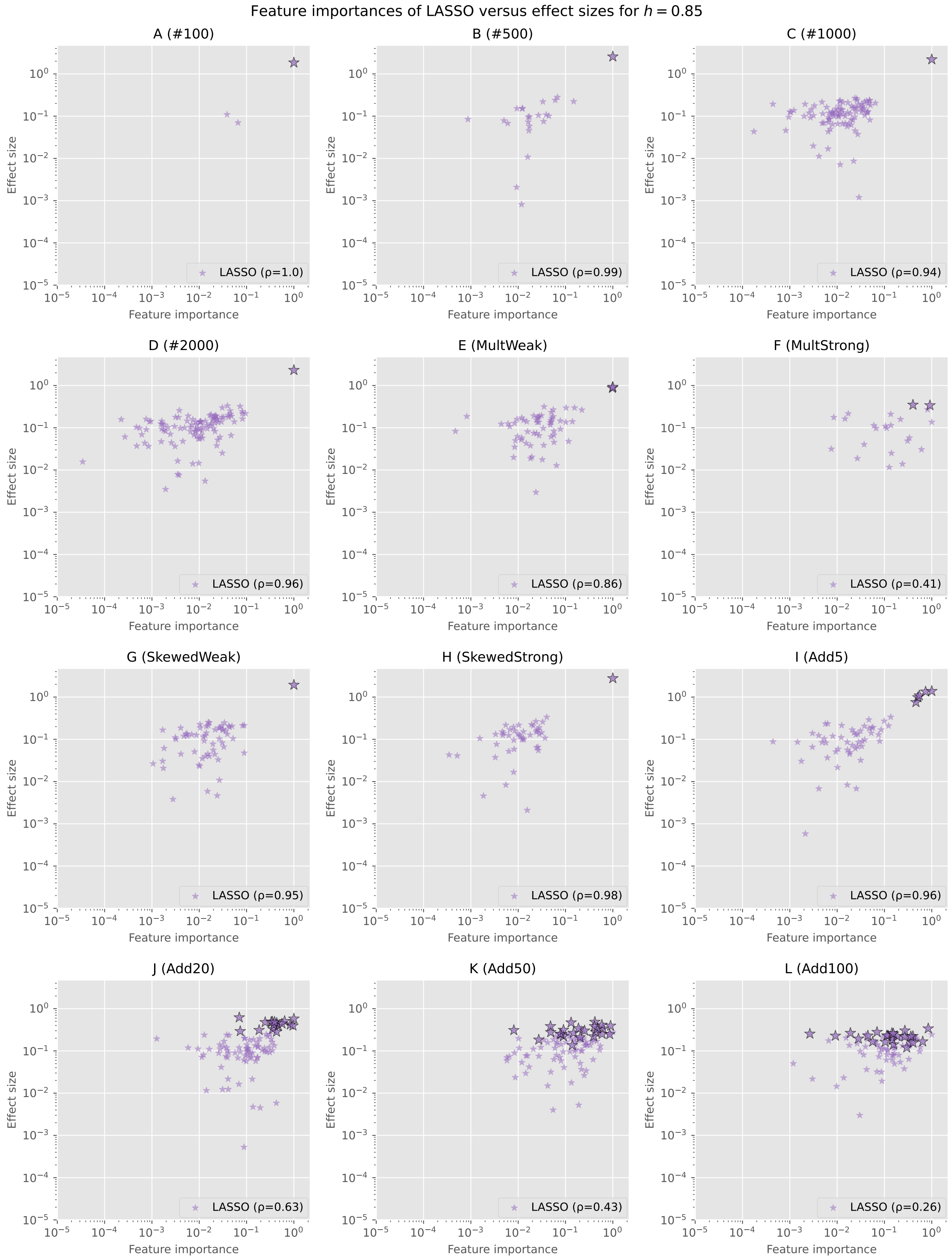

Figure S14. Min-max normalized feature importances of LASSO with effect sizes on synthetic data for $h = 0.85$: Each subplot shows the results of one of the simulation configurations on a logarithmic scale. Only SNPs for which both the effect size as well as the feature importance are not zero are shown. Causal SNPs are highlighted by a larger marker size and a black frame. The legend additionally gives the Pearson correlation coefficient of the effect sizes and feature importances.

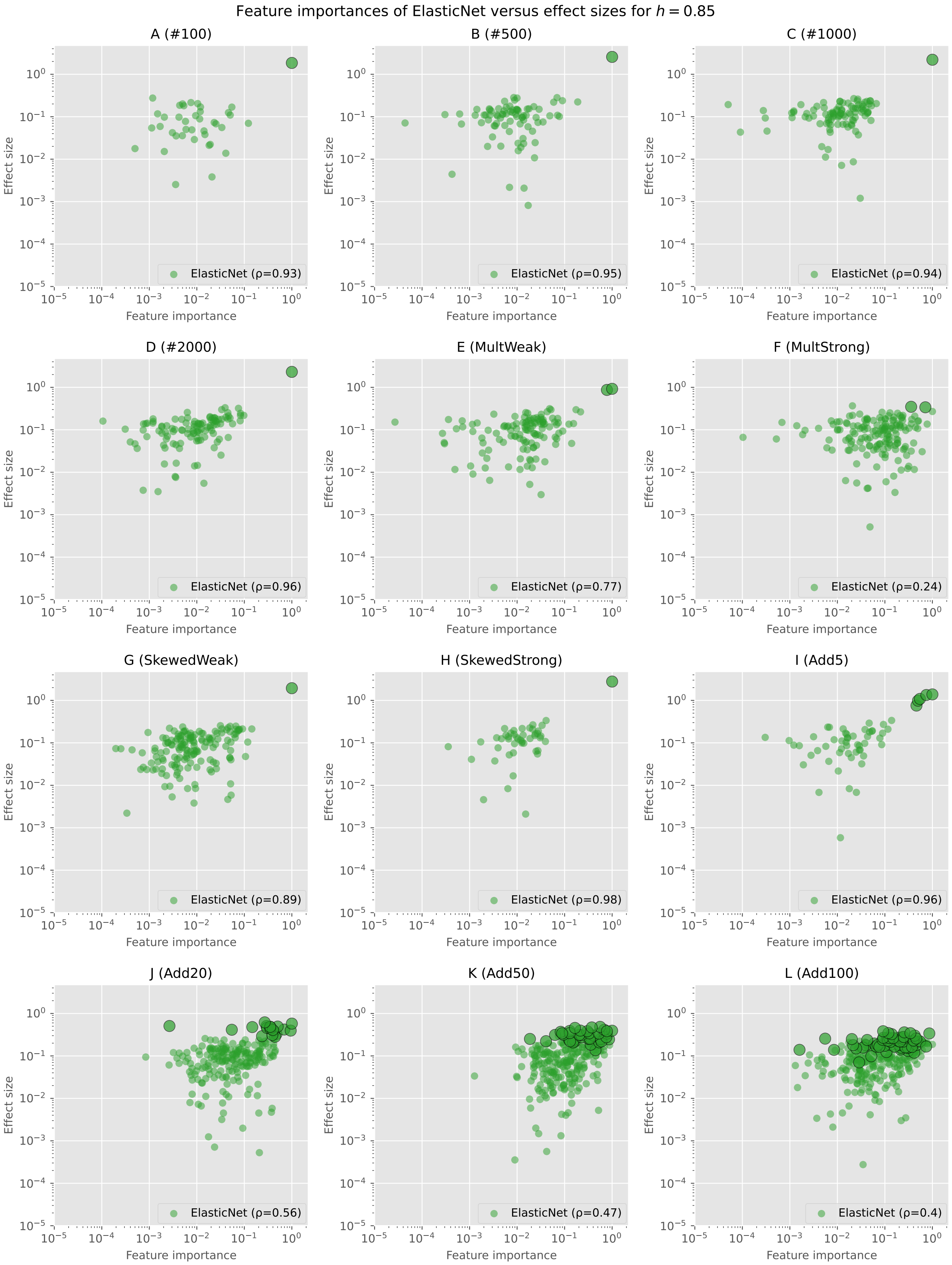

Figure S15. Min-max normalized feature importances of ElasticNet with effect sizes on synthetic data for $h = 0.85$: Each subplot shows the results of one of the simulation configurations on a logarithmic scale. Only SNPs for which both the effect size as well as the feature importance are not zero are shown. Causal SNPs are highlighted by a larger marker size and a black frame. The legend additionally gives the Pearson correlation coefficient of the effect sizes and feature importances.

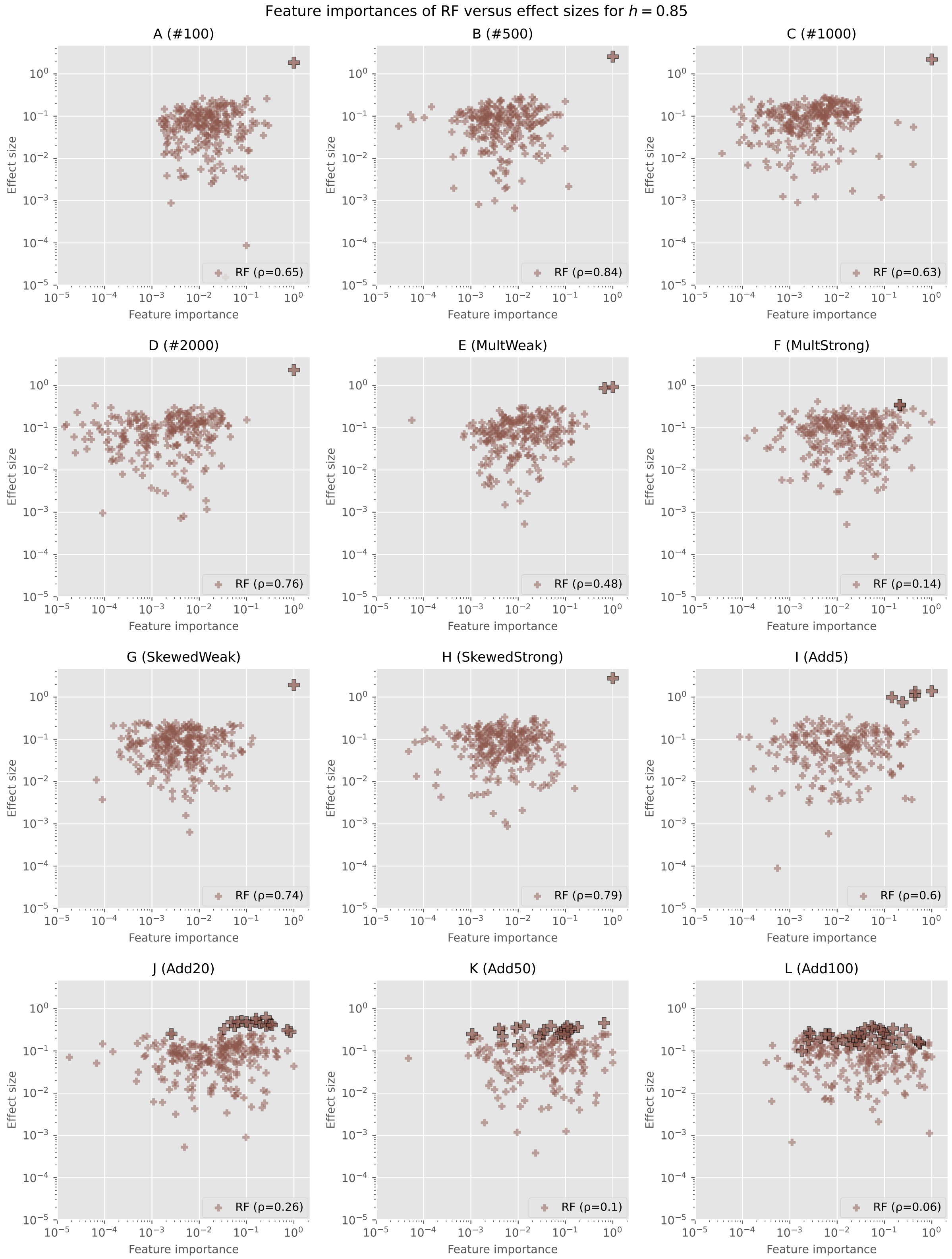

Figure S16. Min-max normalized feature importances of RF with effect sizes on synthetic data for $h = 0.85$**:** Each subplot shows the results of one of the simulation configurations on a logarithmic scale. Only SNPs for which both the effect size as well as the feature importance are not zero are shown. Causal SNPs are highlighted by a larger marker size and a black frame. The legend additionally gives the Pearson correlation coefficient of the effect sizes and feature importances.

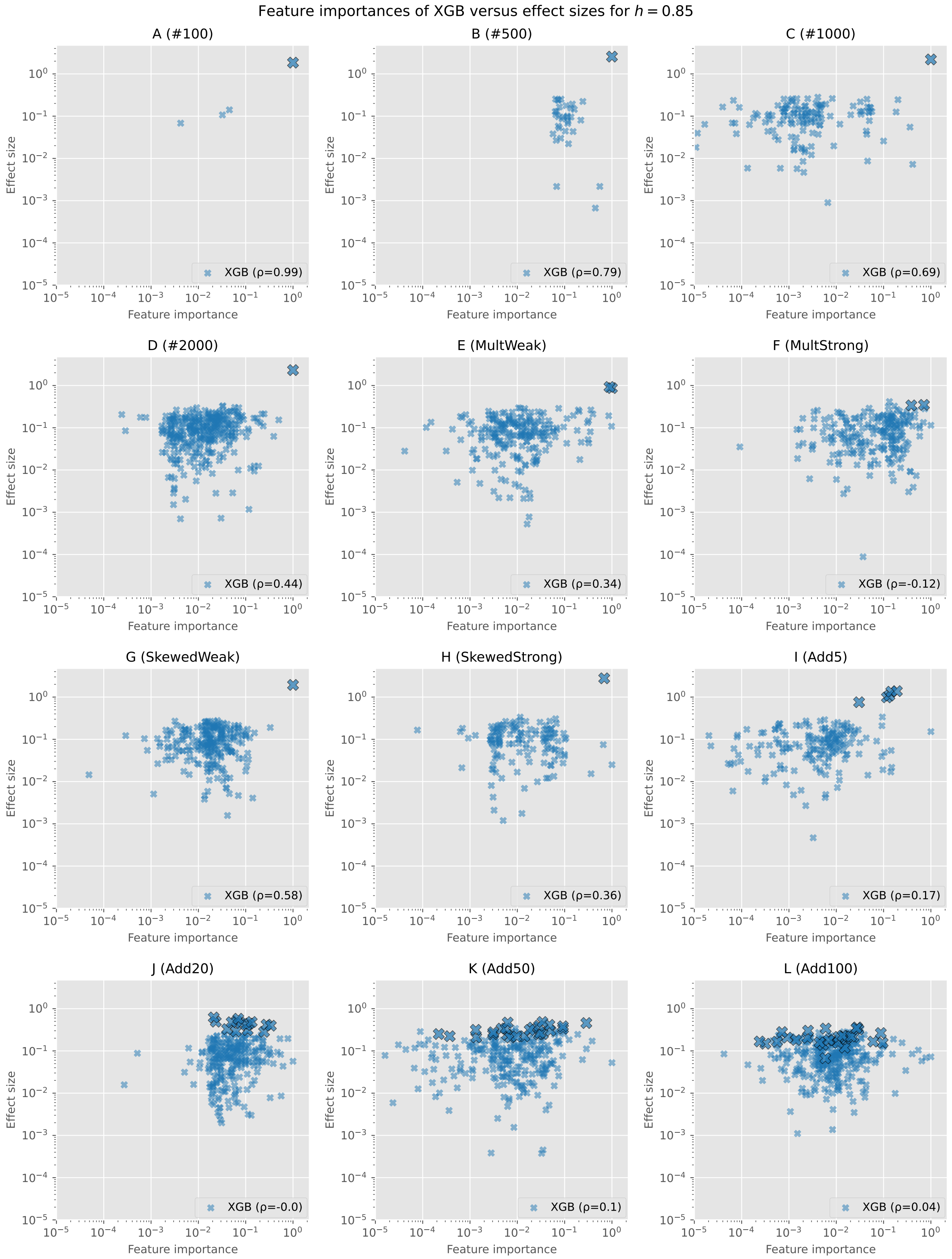

Figure S17. Min-max normalized feature importances of XGB with effect sizes on synthetic data for $h = 0.85$: Each subplot shows the results of one of the simulation configurations on a logarithmic scale. Only SNPs for which both the effect size as well as the feature importance are not zero are shown. Causal SNPs are highlighted by a larger marker size and a black frame. The legend additionally gives the Pearson correlation coefficient of the effect sizes and feature importances.

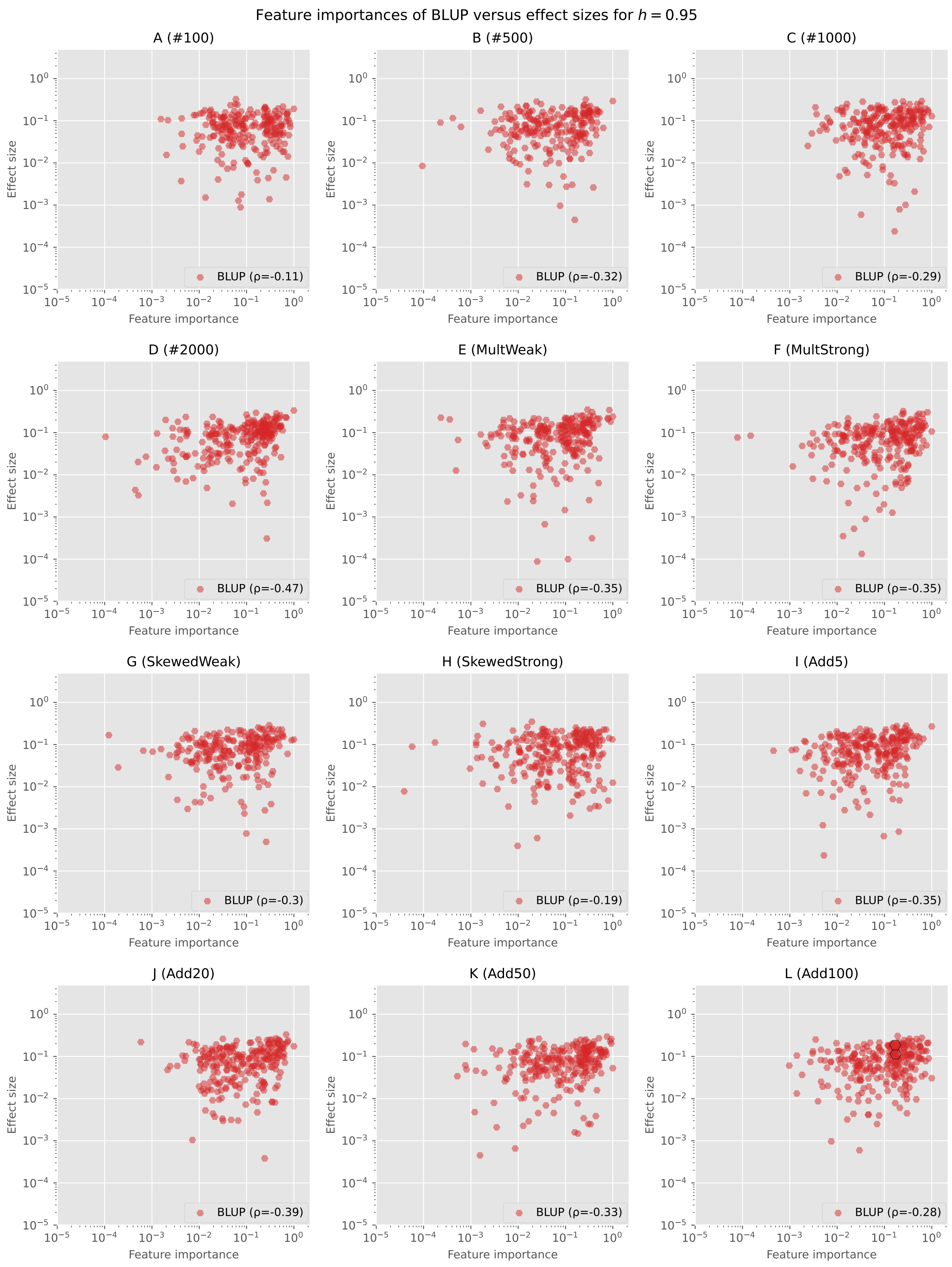

Figure S18. Min-max normalized feature importances of RR-BLUP with effect sizes on synthetic data for $h = 0.95$: Each subplot shows the results of one of the simulation configurations on a logarithmic scale. Only SNPs for which both the effect size as well as the feature importance are not zero are shown. Causal SNPs are highlighted by a larger marker size and a black frame. The legend additionally gives the Pearson correlation coefficient of the effect sizes and feature importances.

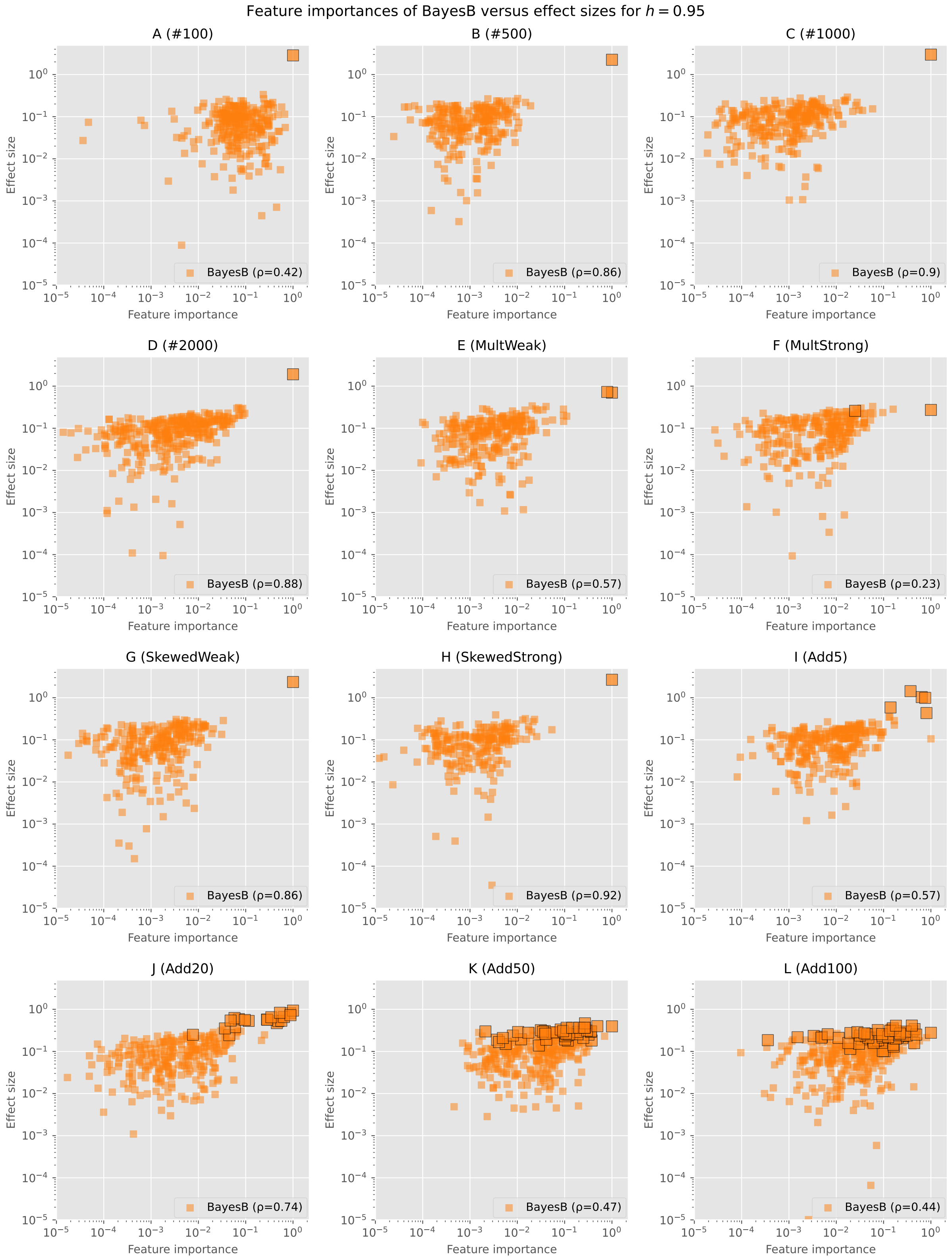

Figure S19. Min-max normalized feature importances of BayesB with effect sizes on synthetic data for $h = 0.95$: Each subplot shows the results of one of the simulation configurations on a logarithmic scale. Only SNPs for which both the effect size as well as the feature importance are not zero are shown. Causal SNPs are highlighted by a larger marker size and a black frame. The legend additionally gives the Pearson correlation coefficient of the effect sizes and feature importances.

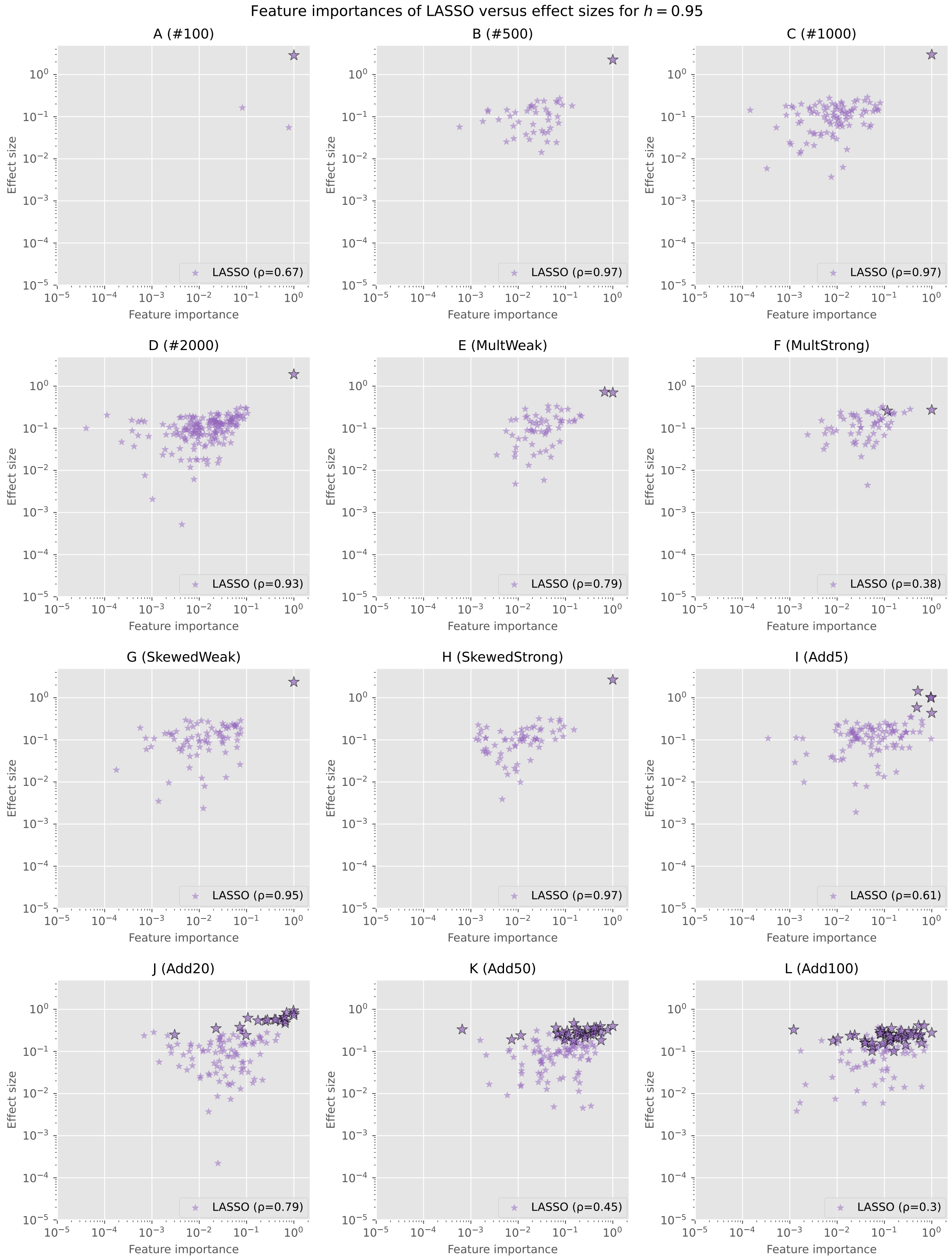

Figure S20. Min-max normalized feature importances of LASSO with effect sizes on synthetic data for $h = 0.95$: Each subplot shows the results of one of the simulation configurations on a logarithmic scale. Only SNPs for which both the effect size as well as the feature importance are not zero are shown. Causal SNPs are highlighted by a larger marker size and a black frame. The legend additionally gives the Pearson correlation coefficient of the effect sizes and feature importances.

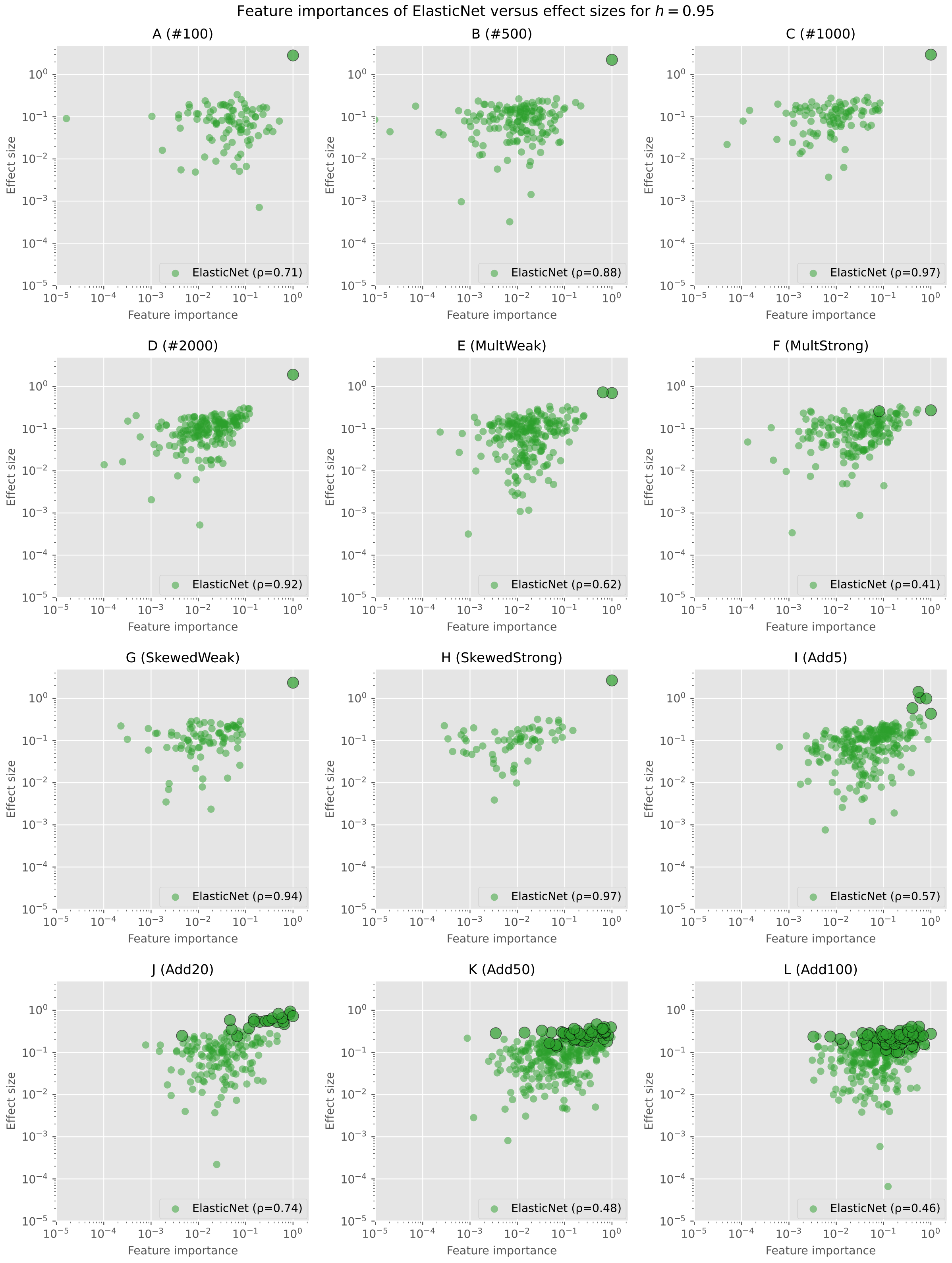

Figure S21. Min-max normalized feature importances of ElasticNet with effect sizes on synthetic data for $h = 0.95$: Each subplot shows the results of one of the simulation configurations on a logarithmic scale. Only SNPs for which both the effect size as well as the feature importance are not zero are shown. Causal SNPs are highlighted by a larger marker size and a black frame. The legend additionally gives the Pearson correlation coefficient of the effect sizes and feature importances.

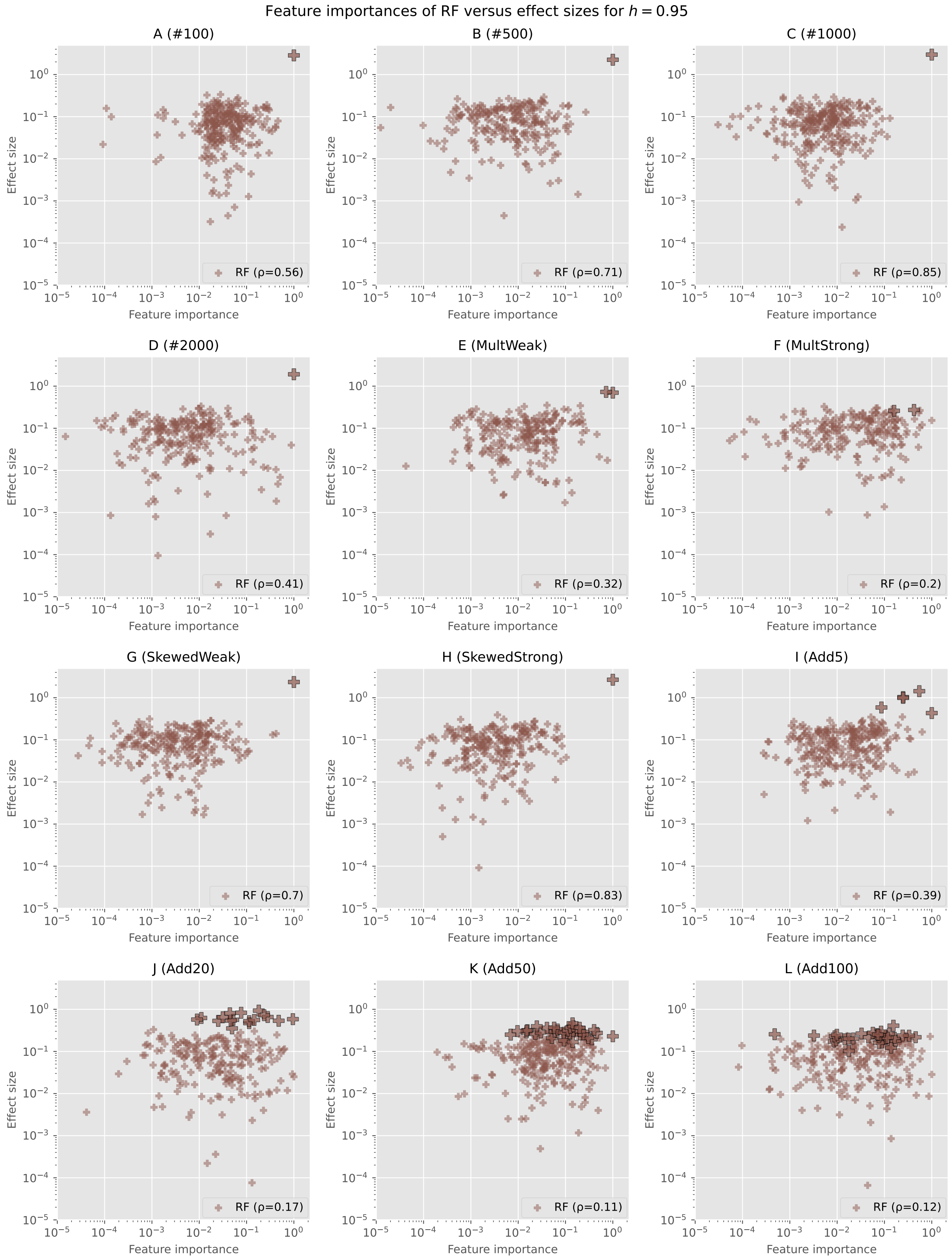

Figure S22. Min-max normalized feature importances of RF with effect sizes on synthetic data for $h = 0.95$: Each subplot shows the results of one of the simulation configurations on a logarithmic scale. Only SNPs for which both the effect size as well as the feature importance are not zero are shown. Causal SNPs are highlighted by a larger marker size and a black frame. The legend additionally gives the Pearson correlation coefficient of the effect sizes and feature importances.

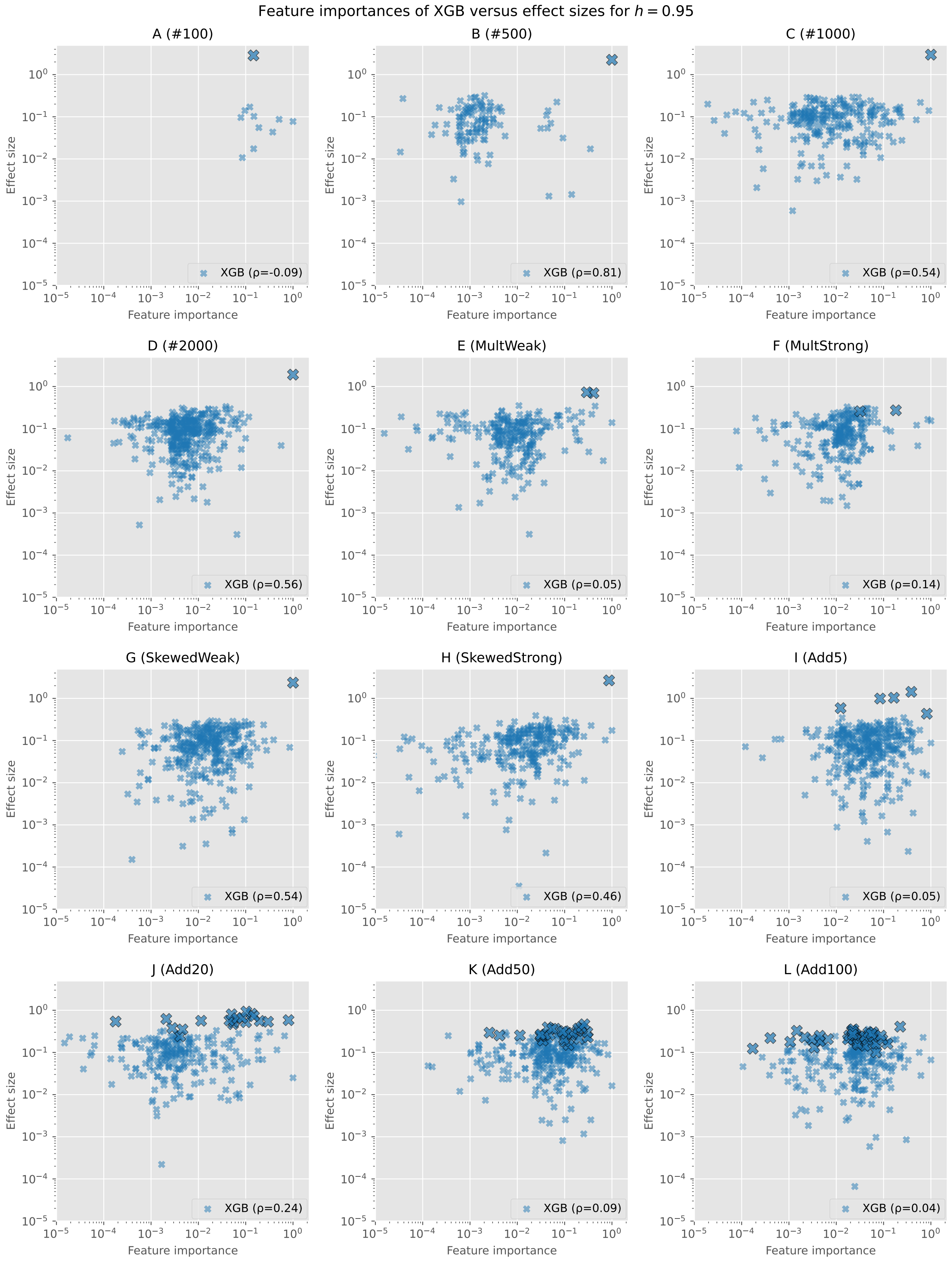

Figure S23. Min-max normalized feature importances of XGB with effect sizes on synthetic data for $h = 0.95$: Each subplot shows the results of one of the simulation configurations on a logarithmic scale. Only SNPs for which both the effect size as well as the feature importance are not zero are shown. Causal SNPs are highlighted by a larger marker size and a black frame. The legend additionally gives the Pearson correlation coefficient of the effect sizes and feature importances.

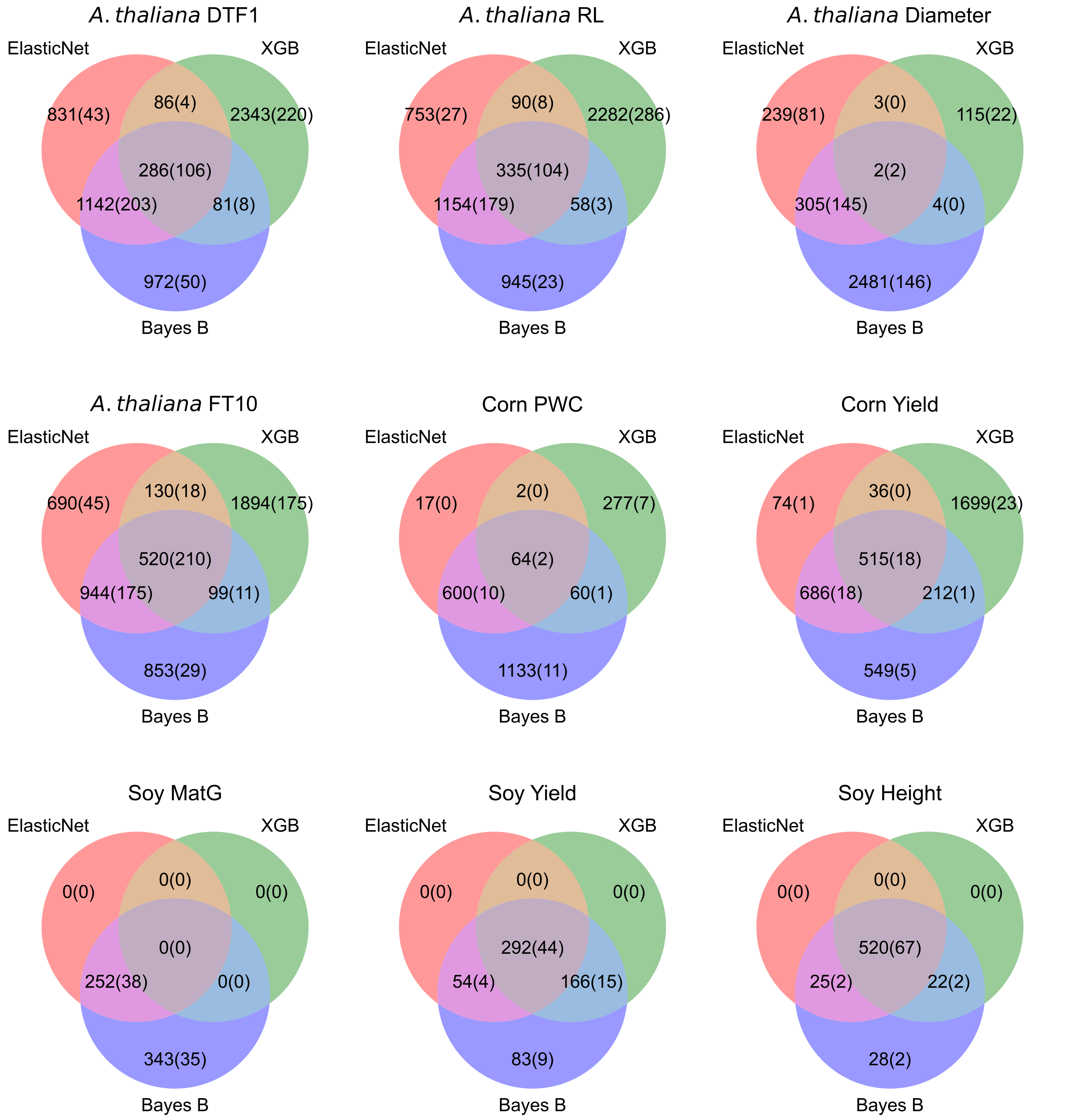

Figure S24. Comparison of feature importance of ElasticNet, Bayes B and XGB for all real-world phenotypes: Each subplot shows the number of important SNPs for the respective model. A SNP is considered important if its related model parameter differs from zero in at least one outer fold of the nested cross-validation. In parenthesis the number of markers is shown which were also among the top 1 000 GWAS results for *Arabidopsis thaliana*, respective top 100 GWAS results for corn and soy.

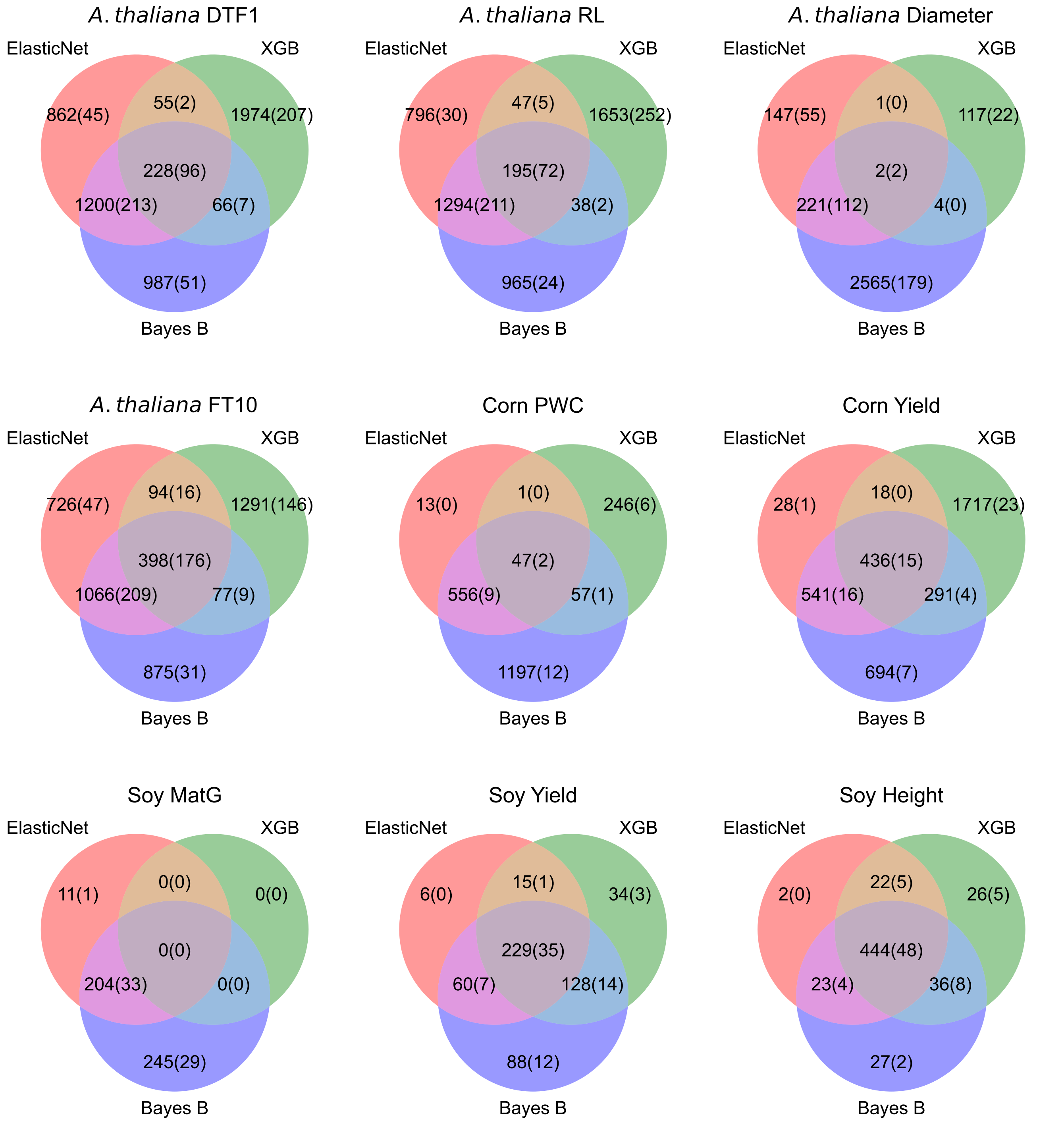

Figure S25. Comparison of feature importance of ElasticNet, Bayes B and XGB for all real-world phenotypes with filtering out those with an importance less than one percentage of the largest value: Each subplot shows the number of important SNPs for the respective model after removing those features that are smaller than one percent of the largest feature importance. A SNP is considered important if its related model parameter differs from zero in at least one outer fold of the nested cross-validation. In parenthesis the number of markers is shown which were also among the top 1 000 GWAS results for *Arabidopsis thaliana*, respective top 100 GWAS results for corn and soy.
